# Structural characterization and thermodynamic behavior of melittin-derived peptide interactions with gram-positive bacterial cell membranes using molecular dynamics simulation

**DOI:** 10.1039/d5ra06511a

**Published:** 2026-03-02

**Authors:** Yosra Delshad, Khaled Azizi, Federico Fogolari, Mokhtar Ganjali Koli

**Affiliations:** a Department of Chemistry, University of Kurdistan Sanandaj Iran k.azizi@uok.ac.ir; b Computational Chemistry Laboratory, Kask Afrand Exire Ltd Sanandaj Iran; c Dipartimento di Scienze Matematiche Informatiche e Fisiche (DMIF), University of Udine Via delle Scienze 206 33100 Udine Italy; d Department of Energy, Politecnico di Torino Corso Duca degli Abruzzi 24 Torino 10129 Italy

## Abstract

This study examined the interaction of different concentrations of a melittin-derived antibacterial peptide with Gram-positive bacterial membranes using molecular dynamics simulations. To achieve a more biologically representative model, the bacterial membrane composition was constructed using nine distinct phospholipid types. The results indicate that in the single-peptide system, the peptide integrates into the membrane and predominantly adopts an α-helical structure. However, in the tetrameric peptide system, due to peptide self-assembly, the peptide effect is exerted through a significant enhancement of electrostatic interactions between the peptides and the membrane surface. This interaction induces structural disorder and surface depressions within the membrane while reducing the migration tendency of sodium ions and water molecules toward the phosphate region. Additionally, the α-helical structure is preserved approximately 4% more in the tetrameric system compared to the single-peptide system. Furthermore, it was determined that arginine residues, together with phosphatidylglycerol-type phospholipids, play the most significant roles in facilitating electrostatic interactions and establishing hydrogen bonds between the peptides and phospholipids. The peptide noticeably reshapes membrane dynamics by reducing lipid mobility in a dose-dependent manner. This effect arises mainly from electrostatic interactions and localized peptide–lipid clustering, which trigger distinct responses across the nine phospholipid species and collectively contribute to greater membrane ordering. As peptide concentration increases, the bilayer becomes more rigid, consistent with enhanced clustering at the membrane surface. Relative shape anisotropy analysis further showed that the single peptide predominantly adopts compact, spherical conformations, whereas tetrameric peptides shift toward more extended, linear, and cylindrical shapes.

## Abstract 2

MDP1–membrane interactions at different concentrations induce localized disruption in Gram-positive bacterial bilayers.

## Graphical abstract

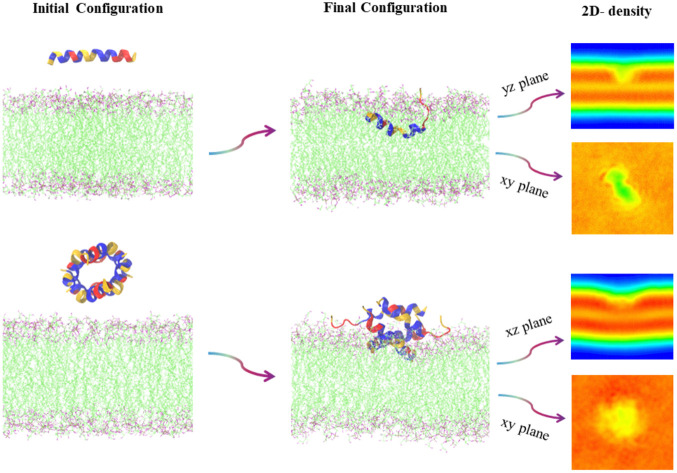

## 1. Introduction

Gram-positive bacteria with relatively thick cell walls cause many infectious diseases.^1^ Among these bacteria, *Staphylococcus aureus* can be mentioned, as it is found in the nasal cavity of warm-blooded animals and can cause severe infections in human tissues, such as blood infections.^2^ The drug resistance of these pathogens, on one hand, and the increase in human–animal interaction, on the other hand, have led to extensive research devoted to their study in recent years.^1,3^

Studies have shown that the composition of the lipid membrane is a key factor in the level of drug resistance and permeability of bacteria.^4^ The cell wall of Gram-positive bacteria is composed of peptidoglycan and lipoteichoic acid, which maintain the integrity of the cell and give the outer surface of the membrane a negative charge.^5,6^ Due to this characteristic, drugs with a positive charge are considered suitable for combating these bacteria.

It has come to light that all living organisms, including plants, possess small, positively charged peptides that have antimicrobial properties.^7,8^ These antimicrobial peptides (AMPs) have the ability to combat bacteria, including Gram-positive bacteria, and prevent bacterial drug resistance.^6^ Research has shown that Pexiganan, MU1140, NISIN, KLR, Bofurin, KR12, and Colicin E1,^5,8–10^ among others, can penetrate the bacterial membrane through anionic adsorption, creating pores and ion channels that disrupt the membrane's integrity, ultimately halting bacterial growth and leading to cell lysis.^11^

Although electrostatic forces and polarity are the main factors in peptide–membrane interactions for these drugs, it has been found that increasing the electrical charge or polarity of most peptides does not necessarily increase their ability to disrupt membranes.^9,12^ Their action also depends on other factors such as the degree of hydrophobicity, secondary structure, and amino acid chain length of the peptide.^8^ This complexity in the mechanism of action has made experimental studies on AMPs ambiguous and inconclusive, with limited information obtained on their effective intermolecular interactions.

As a result, to address the constraints of experimental methods, scientists have progressively shifted to molecular dynamics (MD) simulations to gain deeper insights into the mechanisms and mode of action of AMPs on bacterial membranes.^6,7,13^ These computational analyses have uncovered important insights that enhance and confirm experimental results.

Maleš *et al.* explored how the antimicrobial peptide Adepantine 1 interacts with bacterial membranes composed of POPG and POPE using MD simulations. Their results highlighted the key role of peptide self-assembly in conferring antibacterial activity. Notably, computational data agreed well with experimental observations, showing that Adepantine 1 has very high selectivity against *Escherichia coli* and causes significantly less hemolysis than many other antimicrobial peptides.^6^ In another MD study, Mirnejad *et al.*^7^ investigated the peptide CM11 as known for its potent activity against multidrug-resistant pathogens such as *Staphylococcus aureus* and *Escherichia coli*. Simulations showed that electrostatic interactions are the main driving force in the initial stage of CM11 adsorption to the bacterial membrane surface, while van der Waals and electrostatic forces both contribute to the stability of secondary interactions and also to facilitating the entry of the peptide into the lipid bilayer. Furthermore, Lee *et al.* found that the amino acid arginine plays a vital role in β-Defensin-3's antibacterial effectiveness,^14^ while Herrera-León *et al.* showed that, after crossing the membrane, the alpha-helical peptide HB43 forms a salt bridge between its lysine residue and the phosphate group of the lipids.^13^

Jahangiri *et al.*^15^ demonstrated that in GF-17, the amino acids valine, phenylalanine, and isoleucine regulate peptide assembly on the membrane surface, while arginine and lysine residues drive the peptide-bacterial membrane interaction. Importantly, the aforementioned computational findings have been confirmed by experimental studies; these studies show that the GF-17 peptide has the ability to inhibit bacterial adhesion, prevent biofilm formation, and exhibit bactericidal activity in both Gram-negative bacteria such as *Escherichia coli* K-12 and Gram-positive bacteria including methicillin-resistant *Staphylococcus aureus* (MRSA) strains.^16,17^ Additionally, Khavani *et al.*^18^ showed that alpha-helical peptides such as magainin-2, due to their greater hydrophobicity, penetrate the membrane more efficiently than beta-sheet structures such as protegrin-1 and form more stable bonds with lipid bilayers. These studies collectively demonstrate that MD simulations not only elucidate molecular mechanisms at the atomic level but also guide experimental design and validate therapeutic efficacy.

Building on these insights from MD studies, melittin, which is extracted from honey bee venom,^19^ has been the subject of extensive research through both experimental^19,20^ and computational^21,22^ approaches. Although this peptide demonstrates notable antibacterial^22^ and anticancer^23^ activities, it also has some cytotoxic effects that could affect its therapeutic applications.^24^ Therefore, Akbari *et al.* developed two melittin-derived peptides (MDP1 and MDP2).^24,25^ These peptides demonstrated increased antibacterial features and reduced toxicity towards normal cells. Given that the hemolytic activity of MDP1 is approximately 100-fold lower than that of melittin, MDP1 has been selected for further investigations.

Despite favorable experimental findings in MDP1 studies, the absence of molecular-level insights regarding the dynamics and thermodynamics of the peptide–membrane system has left these studies partially inconclusive. MD simulations provide enhanced clarity in observing biomolecular processes such as reciprocal changes between the drug and the membrane as a potent and reliable technique.^26,27^ By analyzing the thermodynamic and structural properties of the membrane–peptide system, MD simulations can reveal conformation changes, binding interactions, folding/unfolding dynamics, orientation of all atoms, and eventually MDP1's mode of action and the mechanism by which it disrupts membrane integrity.^28^ This understanding is essential for the rational design of effective therapeutic agents and serves as a powerful tool to predict the behavior of novel peptides within biological membranes, thereby accelerating the drug discovery pipeline and improving translational outcomes.^29^

## 2. Computational methods

### 2.1 Peptide structure and membrane composition

The MDP1 peptide consists of 23 amino acids, featuring a hydrophobic N-terminus and a hydrophilic C-terminus. To develop MDP1 with five positive charges, melittin was modified by removing two hydrophobic amino acids, W_19 and I_20, as well as an amino acid, S_18, which was incapable of penetrating the membrane. These modifications yielded the peptide sequence GIGAVLKVLTTGLPALIKRKRQQ.

Experimental characterizations of MDP1 have already confirmed its α-helical secondary structure (*via* CD spectroscopy) and its membrane-disruptive and antibacterial activity (through fluorescence leakage and electron microscopy).^25,30^ According to Dashtbin *et al.* studies, truncating the hydrophobic region decreases peptide toxicity while significantly enhancing antibacterial efficacy as demonstrated in laboratory assays.^31,32^ However, the molecular mechanisms underlying its interaction with bacterial membranes remain unclear due to the lack of atomistic studies. In order to examine the effect of drug concentration and to obtain a clearer understanding of the mechanism underlying the interaction between MDP1 and the membrane, we investigated both single and tetrameric peptide systems to explore the potential role of peptide–peptide interactions in modulating membrane disruption behavior. This atomic-level perspective provides fundamental insights into the molecular basis of the peptide's antibacterial activity.

In this study, MD simulations were carried out to investigate the mechanism of drug interactions with lipid bilayer membranes. It was assumed that the primary amino acid arrangement in the melittin-derived MDP1 was preserved as it self-assembles in aqueous solution into a tetramer, resembling a hashtag (PDB ID in RCSB: 2MLT).^33^ A 4-membered structure of MDP1 was designed through homology modeling^34,35^ by modification of the melittin PDB file obtained from X-ray diffraction observations^33^ and the interaction of the MDP1 peptide with the Gram-positive bacterial membrane has been studied from a molecular perspective. In the current research, the simulated bacterial membrane was designed to be similar to actual membrane by incorporating more phospholipid variety comprising nine distinct phospholipids. Besides, in order to evaluate the effect of concentration on medication performance, MD calculations were repeated to investigate the interaction between a MDP1 tetramer (4MDP1) and the Gram-positive bacterial membrane.

### 2.2 Simulation details

**Fig. 1:**
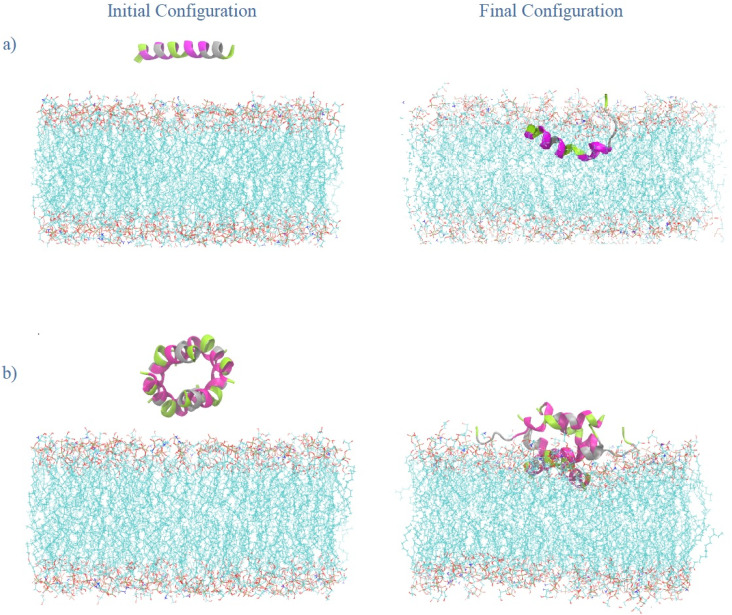
Initial and final configurations of the peptides towards the bacterial membrane for (a) 1MDP1 system, and (b) 4MDP1 system in replica 1.

In this study, nine molecular systems were constructed to evaluate the interaction of MDP1 with a bacterial membrane. A membrane-only model containing the phospholipid bilayer, water, and ions served as the reference, as shown in Table 1 and Fig. S1.^36^ To examine peptide interactions, three independent replicas were generated for a system containing a single MDP1 molecule (1MDP1), each initiated from a distinct peptide orientation. Similarly, three replicas were prepared for a tetrameric MDP1 arrangement (4MDP1) by varying the initial peptide configurations, as illustrated in Fig. 1, S2 and S3. In addition, two peptide-only simulations (one with a single peptide and the other with the tetramer) were performed in bulk water to characterize intrinsic peptide dynamics and to assess the stability of the hashtag-like configuration of the 4MDP1 system in the absence of the membrane. As shown in Movie S1, Table S1 and Fig. S4, the hashtag-like configuration remained remarkably stable throughout the 200 ns production simulation.

**Table 1:** Phospholipid composition in cell membranes of Gram-positive bacteria

Phospholipid name	Number of atoms	Charge	Phospholipids in each leaflet
AIPE	115	0	11
AIPG	117	−1	27
DPPE	121	0	3
DPPG	123	−1	7
MAIPE	112	0	9
MAIPG	114	−1	22
PAIPE	118	0	4
PAIPG	120	−1	9
PAICL	226	−2	8

The initial position of the monomeric peptide in replica 1 was aligned parallel to the membrane and placed at a distance slightly greater than 1.2 nm (a bit more than the cutoff radius) from the membrane surface to prevent any artificial interactions. The same approach was applied in replicas 2 and 3 of the monomeric system, with the difference that in replica 2, the N-terminal end of the peptide was tilted upward by 45°, while in replica 3, the C-terminal end was tilted upward at the same angle, as shown in Fig. S2. In the tetrameric systems, the initial structure of replica 1 was arranged in a pattern resembling the hashtag-like, inspired by the tetrameric configuration of the melittin (as the parent peptide).^33^ Replica 2 was generated by applying a 45° rotation around *y* axis and replica 3 by a 90° rotation around the *x* axis. The initial simulation box dimensions were 8.16 × 8.16 × 10.2 nm^3^ for the monomeric systems, containing approximately 13 000 water molecules. For the tetrameric systems, the box size was 9 × 9 × 11.5 nm^3^, including about 20 000 water molecules.

The TIP3P water model was used to solvate the systems,^37^ and sufficient amounts of sodium and chloride ions were added to each system to achieve a salt concentration of 0.15 M, which corresponds to physiologically relevant ionic conditions resembling those under which cells naturally operate.^38^ Peptide/membrane systems for various simulations were constructed using CHARMM-GUI.^39–46^ The CHARMM36 forcefield was used for the MDP1 and the lipid components of membrane.^47,48^ All simulations were performed using the GROMACS 2022 package.^49–51^ The Nose–Hoover thermostat^52^ was used to keep the temperature steady at 310.15 K, with a coupling time of 0.5 ps. The Parrinello–Rahman barostat^53^ was employed to maintain a constant pressure of 1 bar, with a coupling time constant of 2 ps. Semi-isotropic pressure coupling was applied with two degrees of freedom, one in the *x* and *y* direction and another in the *z* direction and periodic boundary conditions were applied along all axes of the simulation box. The LINCS algorithm was used to constrain all atom bond lengths.^54^ The leap-frog algorithm was used to integrate Newton's equations of motion with a time step of 2 fs.^55^ Electrostatic and vdW interactions were cut-off at 1.2 nm, and the Particle Mesh Ewald method was used for long-range electrostatic interactions.^56^ Unfavorable atomic contacts were removed by steepest descent energy minimization. In the beginning, the system was brought to equilibrium in two NVT and four NPT ensembles (as detailed in SI, page 9). After these equilibration procedures, each system was simulated in the NPT ensemble for 1200 ns. Statistical uncertainties were estimated by block averaging using the GROMACS command gmx analyze on the equilibrated trajectory segments (600–1200 ns), with each replica analyzed independently.

## 3. Result and discussion

### 3.1 Thermodynamics

#### 3.1.1 Interaction energies

As mentioned above, interactions with peptides can affect the membrane environment and play a fundamental role in the thermodynamics of the system by changing its performance, fluidity, and stability.^57^ Therefore, analyzing the results obtained for the interaction energy can provide valuable information about the mechanism of drug–membrane interaction. It should be noted that, so far, no reliable data have been reported to investigate the effect of using 9 phospholipids in MD calculations to compare current study's findings with them.

The results of the calculations for the 1MDP1 system show an electrostatic interaction energy of −996.09 ± 17.25 kJ mol^−1^ (62.2%) and for the vdW −604.81 ± 3.69 kJ mol^−1^ (37.8%). In the 4MDP1 system, the electrostatic interaction energy is −3183.67 ± 69.08 kJ mol^−1^ (85.6%), and for the vdW is −535.76 ± 13.30 kJ mol^−1^ (14.4%). When the number of MDP1 peptides was quadrupled, a 3.19-fold increase in electrostatic interaction energy was noted, which is close to the expected 4-fold increase based on the number of peptides alone. In contrast, the vdW interaction energy actually decreased to 0.89-fold (an 11.4% reduction) instead of showing the expected 4-fold increase. This unexpected decrease in vdW energy suggests that the peptide–membrane interaction is altered when multiple peptides are present, potentially due to reduced available peptide surface area for vdW interactions with the membrane caused by peptide–peptide interactions or self-aggregation. However, other factors such as altered binding geometry, peptide reorientation, or changes in lipid contact composition may also contribute to this observation. The most notable change was the relative contribution from electrostatic interactions, which increased from 62.2% to 85.6%, while the vdW contribution decreased from 37.8% to 14.4%. This shift in contribution balance indicates that the presence of multiple peptides alters the peptide–membrane interaction mechanisms by decreasing the contribution of vdW forces while maintaining strong electrostatic interactions. The first practical implication is that variables enhancing electrostatic interactions may improve the effectiveness of MDP1. This finding is consistent with previous studies that have underscored the importance of electrostatic interactions that aid in the functioning of antimicrobial peptides. Additionally, the concentration-dependent changes in peptide organization result in a relative decrease in vdW contributions towards membrane interaction.

**Fig. 2:**
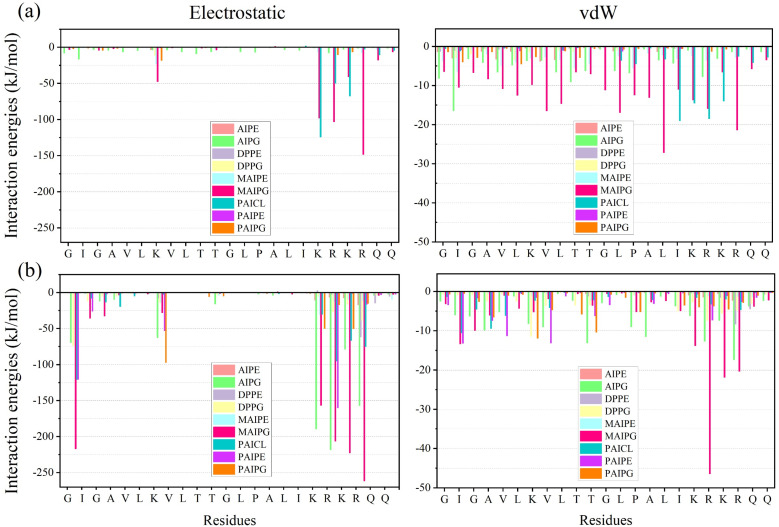
Interaction energies between peptides and different phospholipids for (a) 1MDP1 system, and (b) 4MDP1 system. All results were obtained from the 600–1200 ns interval.

Fig. 2 illustrates the contribution of amino acids to electrostatic and vdW interactions with the membrane. Furthermore, same plots are depicted for replicas in Fig. S5 and S6. Positively charged amino acids from MDP1 are shown to be attracted to the negatively charged membrane by strong electrostatic forces. Table S2 displays the net and normalized energy contribution of each of the nine phospholipids to the total electrostatic and vdW interaction energy as a percentage for the two systems, 1MDP1 and 4MDP1. For 1MDP1, MAIPG and PAICL phospholipids exhibit the highest net contribution to the electrostatic interaction energy at 47.8% and 25.1%, respectively. In vdW interactions, the phospholipids MAIPG and AIPG are once again the most significant contributors to the energy of membrane–drug interaction, accounting for 44.4% and 18.2%, respectively. Additionally, the PAICL phospholipid makes a net contribution of 13.3%.

In terms of molecular structure, MAIPG, which has two hydroxyl groups in its head group and has the shortest carbon chain among charged phospholipids, plays the most significant role in both electrostatic and vdW interactions. Following MAIPG, PAICL with two negative charges contributes most to the membrane–drug interaction. In this regard, the large electric charge and hydrogen bonding agents present in the phospholipid head group may enhance the total energy of drug–membrane interaction. Surprisingly, the arrangement and number of carbon atoms in the hydrocarbon chain of the phospholipid also significantly affects the electrostatic drug–membrane interactions. For instance, the ability of PAIPG, with only 2 more carbon atoms in its carbon tail, to form electrostatic bonds with drugs is less than one-tenth that of MAIPG. This result could be particularly correlated to previous findings indicates that secondary bonds from vdW interactions are also involved in membrane peptide interactions.^7^

The data in Table S2 also shows that for 4MDP1, MAIPG, AIPG, PAIPG, and PAICL phospholipids have the highest net contribution to the electrostatic interaction energy, with 33.5%, 21.8%, 14.5%, and 10.3%, respectively. In terms of the vdW interaction energy, MAIPG and AIPG phospholipids have the highest net contribution, with 32.4% and 15.8%, respectively. Consequently, it is clear that MAIPG phospholipid has a major influence on both electrostatic and vdW energies for 4MDP1, similarly to how it does for 1MDP1.

It's critical to determine whether the number of phospholipids in the membrane or their particular molecular structure is primarily responsible for their contribution to electrostatic and vdW interactions with the drugs. Accordingly, the normalized contribution of vdW and electrostatic interaction energy for each phospholipid is also shown in Table S2. This was calculated by dividing the net energy contribution by the number of that phospholipid present in the membrane. These results indicate that, for the 1MDP1 system, phospholipids with larger normalized values of electrostatic and vdW interaction energies also have a larger net contribution to the total electrostatic and vdW interaction energies, with minor deviations being ignored. Hence, it can be inferred that the primary contribution of MAIPG and PAICL phospholipids to electrostatic interactions arises not only from their abundance in the membrane but also from their distinct molecular structure. The atmosphere for the 4MDP1 system, however, differ from those for the 1MDP1 system. With the exception of MAIPG, the phospholipids exhibiting the highest normalized values of electrostatic and vdW interaction energies differ from those with the largest net contributions to the overall electrostatic and vdW interaction energies. This discrepancy implies that the drug–membrane interaction mechanism within the 1MDP1 system may deviate from that observed in the 4MDP1 system.

In the drug design process, it is crucial to quantify the contribution of each selected amino acid to the drug's overall effectiveness. This can be achieved by analyzing the electrostatic and vdW interaction energies. Accordingly, to identify the amino acids in this study that significantly contribute to the formation of electrostatic or vdW interactions, as well as the specific phospholipids with which they interact most strongly, for all the amino acids of the peptide interactions relevant to drug–membrane interactions were calculated and assessed for both the 1MDP1 and 4MDP1 systems (see Table S5 for results). Based on the data presented in this table for the 1MDP1 system, approximately 54% of the drug–membrane electrostatic interaction energy is attributed to just three interactions: R-MAIPG, K-PAICL, and K-MAIPG. Conversely, these interactions collectively contribute only 24.8% of the electrostatic interaction energy in the 4MDP1 system. Furthermore, the vdW interaction energy of the strongest drug–membrane interaction in the 1MDP1 system (L-MAIPG) is comparable to that in the 4MDP1 system (R-MAIPG).

These observations indicate that the drug MDP1 employs distinct interaction mechanisms with the membrane in the 1MDP1 and 4MDP1 systems. Table S3 presents the electrostatic and vdW interaction energy values for each residue of peptide in the 1MDP1 and 4MDP1 systems. The data reveal that the primary contributors to interaction energy in both systems are either charged residues K-18 to R-21. Interestingly, in the 4MDP1 system, 39.4% of the total electrostatic interaction energy and 22.1% of the total vdW interaction energy were provided by the amino acid arginine. Consistent with previous research,^14^ arginine residues generally offer greater electrostatic stabilization than lysine, likely due to the distributed charge of the guanidinium group reducing desolvation penalties.

We end this section by pointing out that residues utilize more vdW interactions to promote the self-aggregation phenomena in the 4MDP1 system. Moreover, the peptide assumes a configuration that facilitates effective electrostatic interactions between residues and phospholipids, thereby enabling all positively charged amino acids to participate in membrane engagement. This observation warrants further investigation as a potential explanation for the enhanced pharmacological properties of 4MDP1 relative to 1MDP1.

#### 3.1.2 Hydrogen bonds

Assessing the function of hydrogen bonds is crucial for understanding the way peptides interact with phospholipid membranes. This is due to the high probability of hydrogen bond formation in these structures, and hydrogen bonds play a pivotal role in the thermodynamic stability, self-assembly, and formation of secondary structures in peptides and proteins.^58^ As mentioned in previous studies, the main factor in hydrogen bond formation between the peptide and the membrane is the interaction of the hydrogen of the amino group of a residue with the oxygens of the phosphate group of phospholipids.^13^Table 2 displays the number of hydrogen bonds formed between the residues of the MDP1 peptide and each of the membrane phospholipids separately for the two systems, 1MDP1 and 4MDP1. The results in this table indicate that the amino acid arginine, which contains two amino groups in close proximity, plays a significant role in hydrogen bonding interactions by forming bidentate bonds with the phospholipid head groups.^27^ It is important to highlight that the significance of arginine residues in the antibacterial activity of therapeutic peptides has been previously noted. Computational studies of hBD3 have shown that key arginine residues (R_17, R_36, and R_38) contribute to antimicrobial activity through both polar and nonpolar interactions with bacterial membranes, suggesting that their function extends beyond simple electrostatic attraction to include stabilization of the peptide–membrane interface through dual-mode binding.^14^

**Table 2:** The number of hydrogen bonds formed between the residues of the MDP1 peptide and each of the membrane phospholipids for the two systems 1MDP1 and 4MDP1^b^

Simulated systems	Residues^a^	G_1	I_2	K_7	K_18	R_19	K_20	R_21	All residues
	Phospolipids								
1MDP1	DPPG	0.01 (±0.00)	—	0.09 (±0.06)	—	—	0.01 (±0.01)	—	0.13 (±0.09)
	MAIPG	0.07 (±0.02)	0.01 (±0.00)	0.36 (±0.15)	0.8 (±0.14)	1.44 (±0.22)	0.64 (±0.15)	1.64 (±0.18)	5.85 (±0.47)
	AIPE	0.01 (±0.01)	—	0.03 (±0.03)	—	—	—	0.01 (±0.01)	0.06 (±0.03)
	MAIPE	0.03 (±0.02)	0.02 (±0.01)	0.19 (±0.05)	0.05 (±0.03)	0.02 (±0.01)	0.01 (±0.01)	0.05 (±0.04)	0.53 (±0.13)
	PAIPG	0.04 (±0.04)	0.02 (±0.02)	0.13 (±0.07)	—	0.14 (±0.14)	0.06 (±0.05)	—	0.52 (±0.19)
	PAIPE	—	—	0.01 (±0.01)	—	—	—	—	0.01 (±0.01)
	DPPE	0.01 (±0.01)	—	—	—	0.01 (±0.01)	—	—	0.02 (±0.01)
	AIPG	0.19 (±0.03)	0.13 (±0.06)	0.16 (±0.06)	—	0.35 (±0.22)	0.59 (±0.17)	0.03 (±0.02)	2.02 (±0.41)
	PAICL	—	—	—	0.93 (±0.05)	1.51 (±0.38)	0.58 (±0.18)	—	3.27 (±0.54)
	SUM	0.36 (±0.02)	0.17 (±0.05)	0.97 (±0.11)	1.78 (±0.16)	3.49 (±0.11)	1.88 (±0.08)	1.73 (±0.15)	12.40 (±0.31)
4MDP1	4MDP1	G_1	I_2	K_7	K_18	R_19	K_20	R_21	SUM
	DPPG	0.02 (±0.01)	—	0.28 (±0.06)	0.1 (±0.06)	0.39 (±0.19)	0.21 (±0.05)	0.36 (±0.09)	1.59 (±0.26)
	MAIPG	1.51 (±0.20)	0.58 (±0.16)	1.26 (±0.07)	0.45 (±0.07)	2.85 (±0.26)	0.8 (±0.25)	2.42 (±0.42)	11.69 (±1.12)
	AIPE	0.15 (±0.11)	—	0.43 (±0.14)	0.22 (±0.04)	0.29 (±0.06)	0.36 (±0.17)	0.37 (±0.18)	2.41 (±0.64)
	MAIPE	0.41 (±0.16)	0.01 (±0.01)	0.01 (±0.01)	0.31 (±0.04)	1.21 (±0.31)	0.01 (±0.01)	—	2.82 (±0.54)
	PAIPG	0.35 (±0.14)	0.06 (±0.05)	0.85 (±0.17)	0.02 (±0.01)	1.37 (±0.28)	0.38 (±0.15)	1.54 (±0.10)	5.37 (±0.29)
	PAIPE	—	—	—	0.08 (±0.05)	—	—	—	0.09 (±0.05)
	DPPE	—	—	—	—	0.01 (±0.00)	0.14 (±0.03)	0.12 (±0.08)	0.28 (±0.12)
	AIPG	0.37 (±0.10)	0.62 (±0.18)	0.25 (±0.05)	1.2 (±0.19)	2.27 (±0.31)	0.95 (±0.06)	0.98 (±0.30)	7.48 (±0.74)
	PAICL	0.13 (±0.11)	0.09 (±0.09)	0.02 (±0.02)	0.59 (±0.14)	1.55 (±0.19)	0.31 (±0.11)	1.03 (±0.36)	3.88 (±0.58)
	SUM	2.96 (±0.16)	1.36 (±0.13)	3.08 (±0.19)	2.97 (±0.20)	9.94 (±0.53)	3.17 (±0.23)	6.82 (±0.34)	35.6 (±0.91)

a This table only lists amino acids that have formed at least one hydrogen bond with the membrane.

b All results were obtained from the 600–1200 ns interval.

In the 1MDP1 system, the phospholipids MAIPG, PAICL, and AIPG formed most of the hydrogen bonds with the peptide. In the 4MDP1 system, in addition to these three phospholipids, the phospholipid PAIPG formed a substantial number of hydrogen bonds with the peptide, increasing its total hydrogen bond count from approximately zero in the 1MDP1 system to around five in the 4MDP1 system. Furthermore, MAIPG exhibited the highest number of hydrogen bonds in both systems. In the 4MDP1 system, the interaction between MAIPG and R-21 represented the highest number of hydrogen bonds, approximately one and a half times the number observed in the 1MDP1 system. The data in Table 2 shows that the total number of hydrogen bonds for the 4MDP1 system is approximately three times that of the 1MDP1 system. In both systems, the neutral phospholipids did not participate in hydrogen bonding interactions. However, as noted in Section 3.1, the contribution of vdW interactions (including those in hydrogen bonds) to the total drug–membrane binding energy for 4MDP1 system is reduced by 11.4% compared to that of 1MDP1 system. An explanation may be that hydrogen bonds are accounted for in the CHARMM force field by a combination of favorable electrostatic interactions and unfavorable vdW interactions. The stronger the hydrogen bond the less favorable is the vdW interaction.^59,60^

#### 3.1.3 Electrostatic potential

**Fig. 3:**
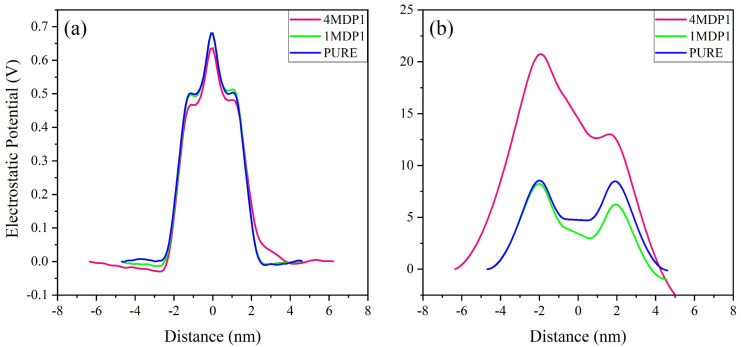
Electrostatic potential profiles along the bilayer normal for (a) system, (b) and Na^+^ ions at different concentration of MDP1. All results were obtained from the 600–1200 ns interval.

A system's behavior can be affected by electrostatic potential, particularly when ions and peptides enter the membrane through ion channels and holes.^61,62^ The electrostatic potential of the system at various distances from the center of the membrane is determined by the Poisson equation:^63^1
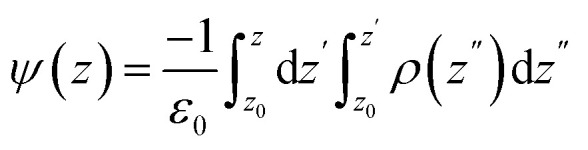
where *ε*_0_ is the vacuum permittivity, *ρ*(*z*) represents the charge density in the *z* direction, *z*_0_ is the reference position where the electrostatic potential is set to zero, which in our calculations this point corresponds to the center of the water region. The electrostatic potential diagram for the entire system and that due to sodium ions are shown in Fig. 3. According to Fig. 3a, the highest electrostatic potential values for the pure membrane systems, 1MDP1, and 4MDP1 appear at 0.681, 0.676, and 0.634 V, respectively. The decrease in the peak height of the 4MDP1 system compared to the peaks of the pure membrane and 1MDP1 indicates a change in the upper membrane leaflet environment for the 4MDP1 system. This change can be attributed to the neutralization of a number of negative membrane charges by the 4 positively charged peptides. While this reduction is within the expected range of numerical variation, it may qualitatively indicate localized charge compensation at the membrane surface.

Another peak is also observed inside the membrane at *x* = 1.1 nm, which is close to the phospholipid head group region. In this region, the peak height is also larger for the 1MDP1 system compared to the 4MDP1 system. This suggests that even if ions can reach the lipid head group region, ion transport in this region is still more difficult for the 4MDP1 system than for 1MDP1. The electrostatic potential diagram of sodium ions shown in Fig. 3b illustrates the change in the membrane environment in both the 1MDP1 and 4MDP1 systems compared to the pure system. Around *x* = 2 nm, which corresponds to the outer surface of the membrane, the height of the sodium peak for the 1MDP1 system is reduced compared to the pure membrane, while for the 4MDP1 system, it is significantly increased.

One other peak is also observed inside the membrane at *x* = 1.1 nm, which is close to the phospholipid head group region. In this region, the peak height is also larger for the 1MDP1 system compared to the 4MDP1 system. This suggests that even if ions can reach the phospholipid head group region, ion transport in this region is still more difficult for the 4MDP1 system than for 1MDP1.

The electrostatic potential diagram of sodium ions shown in Fig. 3b illustrates the change in the membrane environment in both the 1MDP1 and 4MDP1 systems compared to the pure system. Around *x* = 2 nm, which corresponds to the outer surface of the membrane, the height of the sodium peak for the 1MDP1 system is reduced compared to the pure membrane, while for the 4MDP1 system, it is significantly increased. This situation can be attributed to the accumulation of positively charged ions in this region, compared to 1MDP1. This is due to the reluctance of the ions to migrate to the upper leaflet region because the negative charges of the phosphate groups are neutralized by the positive charges of the peptides. Therefore, it can be concluded that in the 4MDP1 system, the change in the electrostatic potential of the membrane makes it more difficult for ions to enter compared to 1MDP1. This may contribute to greater membrane-level disturbances and potentially affect ion-related membrane processes.

### 3.2 Dynamics and structural properties

#### 3.2.1 Membrane-level structural disruption

The lipid component of the cell membrane acts as a dynamic and selective barrier that preserves cellular structural integrity and regulates both intracellular and extracellular homeostasis.^64^ AMPs can eliminate bacteria primarily through membrane disruption. They achieve this by binding to the membrane surface and subsequently forming pores, leading to compromised membrane integrity and cellular death.^65^

**Fig. 4:**
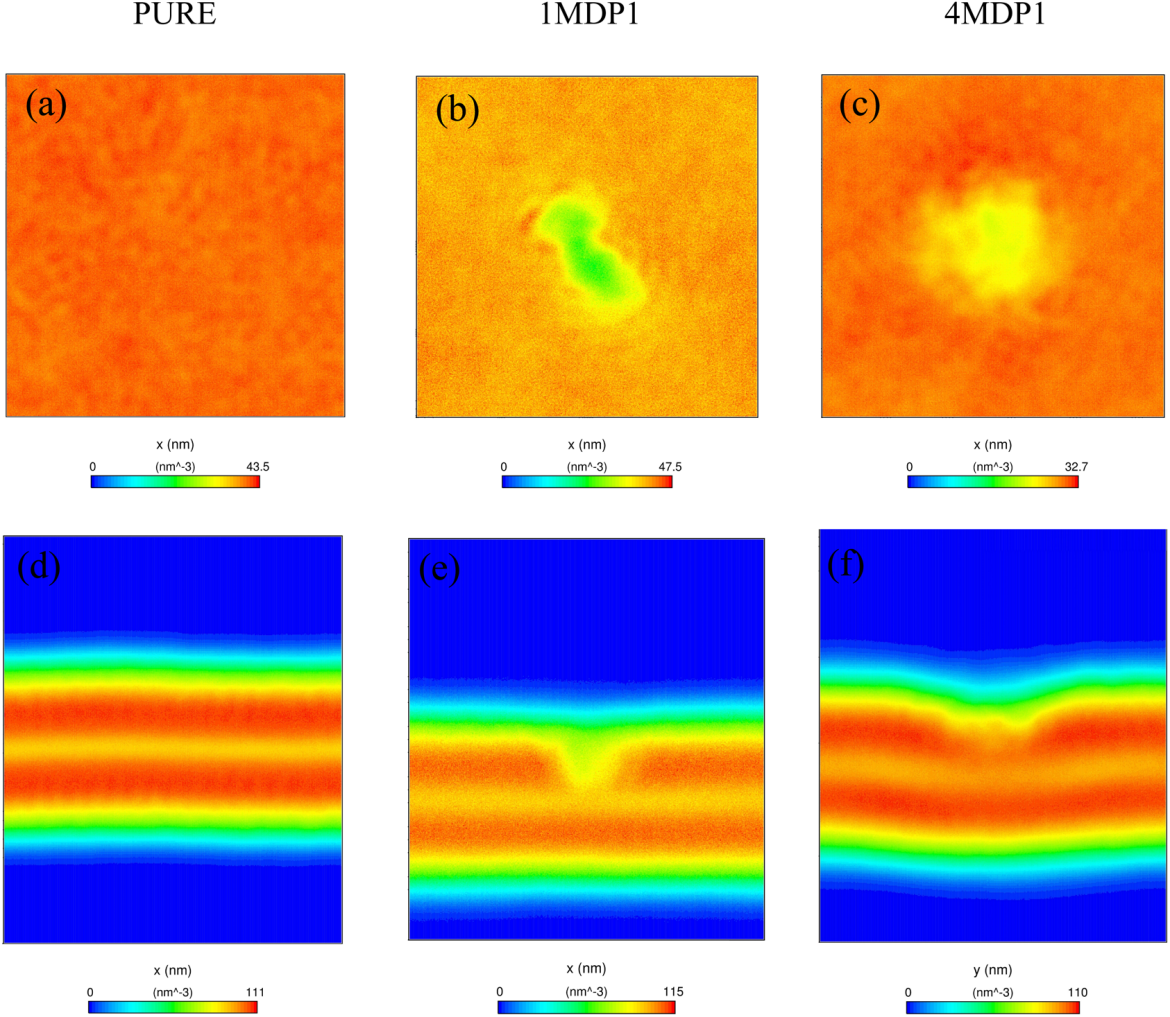
2D-density plots of (a) pure system in *z*-direction, (b) 1MDP1 system in *z*-direction, (c) 4MDP1 system in *z*-direction (d) pure system in *y*-direction, (e) 1MDP1 system in *y*-direction and (f) 4MDP1 system at *x*-direction.

The 2D-density maps depicted in Fig. 4 clearly reveal localized depressions and density voids at the membrane–peptide interface in both 1MDP1 and 4MDP1 systems. In the 1MDP1 system, the observed cavity formation suggests partial peptide insertion into the bilayer, whereas in the 4MDP1 system, surface accumulation of multiple peptides leads to an extended depression consistent with local thinning and destabilization of the upper leaflet. These observations indicate a cooperative perturbation of the bilayer structure that may correspond to the early stages of peptide clustering or membrane destabilization consistent with pore-like behavior.

#### 3.2.2 Electron density

Electron density serves as a key parameter for determining membrane thickness. By analyzing electron density profiles across the membrane, researchers can precisely measure the distance between the peaks corresponding to the lipid headgroups, providing an accurate estimation of the bilayer thickness.^66,67^ The findings demonstrate strong compatibility between the electron density measurements and the experimental data, highlighting the reliability of electron density as a parameter for structural analysis.^68^

**Fig. 5:**
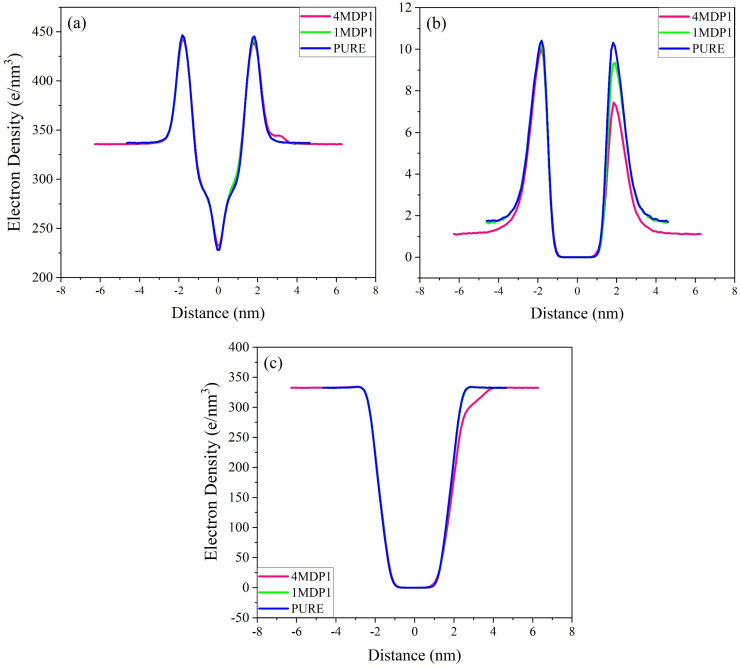
Electron density plots of (a) all atoms, (b) sodium ions and (c) water for pure, 1MDP1 and 4MDP1 system.

Fig. 5 presents the electron density profiles for the pure, 1MDP1, and 4MDP1 systems. In Fig. 5a, two symmetrical peaks at approximately ±1.8 nm are observed, corresponding to the positions of the phosphate groups (P–P distances) in the lipid head regions of the bilayer. The distance between these two peaks, representing the average global membrane thickness, was measured to be 3.66, 3.63, and 3.68 nm in the pure, 1MDP1, and 4MDP1 systems, respectively. Although minor variations are observed, these differences of approximately 0.05 nm are within the statistical uncertainty typical of atomistic MD simulations. Therefore, the bilayer thickness can be considered essentially unchanged across all systems.

This finding indicates that peptide adsorption at the membrane surface does not lead to measurable thinning or thickening of the bilayer, but instead induces only localized electrostatic reorganization without significant structural alteration of the membrane. On the other hand, according to Fig. 5b, the electron density of sodium ions also reaches a maximum value at approximately the same distances. This suggests that the accumulation of sodium ions in the pure membrane and the 1MDP1 and 4MDP1 systems occurs very close to the surface phosphate groups. In the 4MDP1 system, as shown in Fig. 5b, there is a significant decrease in the electron density of sodium. This decrease can be attributed to the neutralization of the negative electrical charges of the phosphate head group as a result of interaction with the positive charges of the peptides, which reduces the tendency of sodium ions to migrate to this area. Additionally, the presence of more peptides near the membrane can act as a barrier, preventing sodium ions from approaching the phospholipid head region. Fig. 5c demonstrates a reduction in the accumulation of water molecules along the outer leaflet in the 4MDP1 system. This phenomenon can be explained by the neutralization of the negative electrical charges of the phosphate head group through interactions with positively charged molecules. Consequently, the tendency of water molecules to migrate to this region is diminished.

### 3.3 Lipid organization

#### 3.3.1 Lipid aggregation

Lipid clustering is another parameter that can be observed in the membrane in the context of drug design. This feature can shed light on how the presence of a drug affects membrane characteristics. To investigate lipid aggregation behavior, cluster analyses were performed focusing on lipids within the upper membrane leaflet. Since clustering is inherently probabilistic, it is essential to consider the statistical population of lipids in the calculations to accurately assess it.

**Fig. 6:**
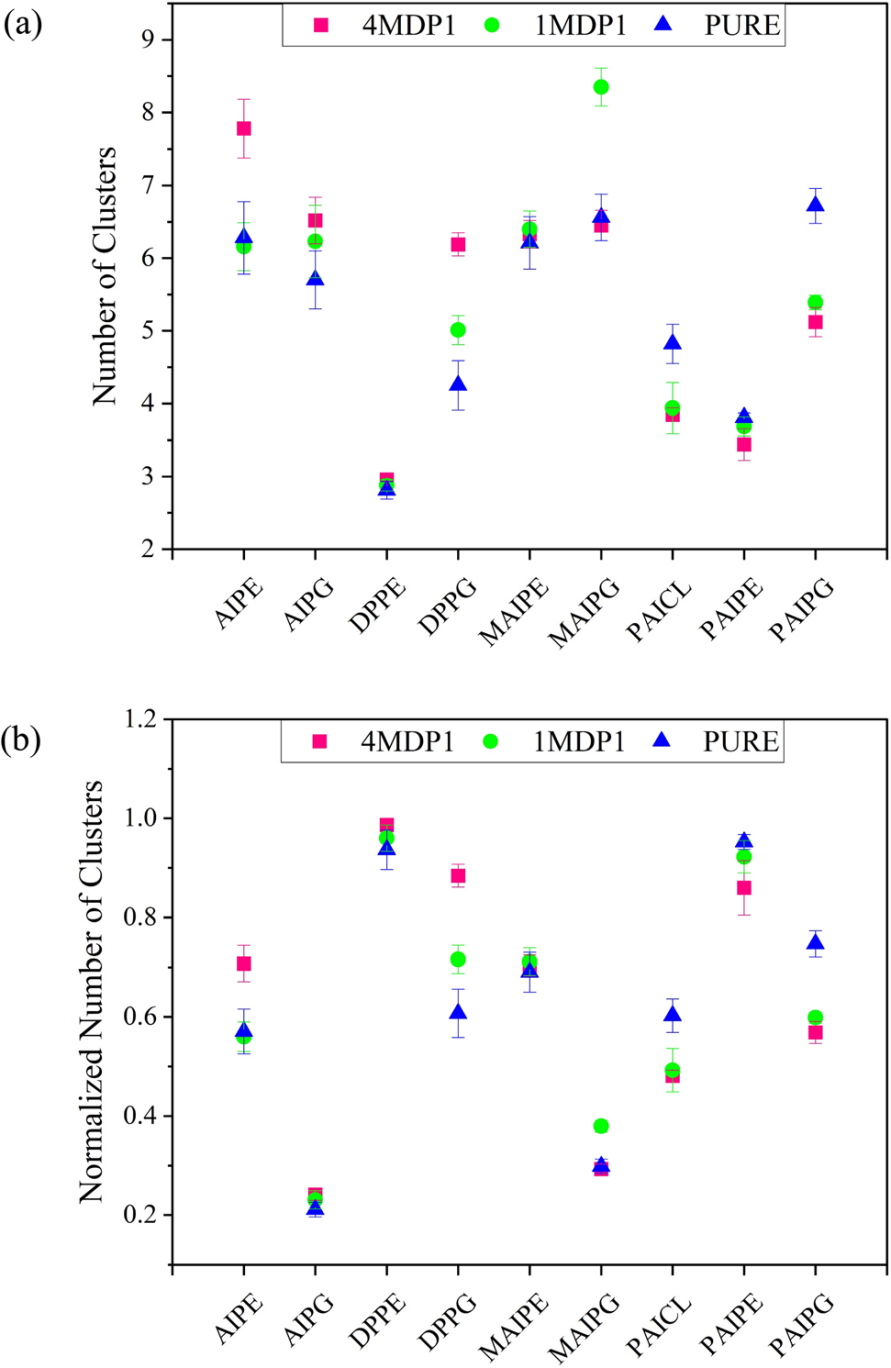
(a) NC for phospholipids in the pure, 1MDP1 and 4MDP1 systems (b) NNC for phospholipids in the pure, 1MDP1 and 4MDP1 systems. All results were obtained from the 600–1200 ns interval accompanied with errors.

In this study, the gmx clustsize command available in GROMACS was used for our clustering procedure. This command implements a distance-based clustering algorithm with a default distance threshold of 3.5 Å. The Normalized Number of phospholipid Clusters (NNC) was calculated and examined by determining the ratio of cluster count to the total number of phospholipids present in the membrane. Fig. 6 displays the number of clusters (NC) and NNC values for membrane phospholipids in their pure state and in the presence of 1MDP1 and 4MDP1. The Fig. 6b shows that, in general, the NNC changed compared to the pure system in both 1MDP1 and 4MDP1 systems, with the effect being more pronounced in the 4MDP1 system. Therefore, it appears that 4MDP1 had a greater impact on the membrane compared to 1MDP1. Additionally, in the pure system, PAIPE and DPPE lipids exhibited the highest NNC values. This observation can be explained by considering the molecular structure of the lipids.

The presence of electrical charge in the phospholipid head region and side chain in its hydrocarbon tail are two key factors that influence clustering. Phospholipids without side chain in their hydrocarbon tail and lacking electrical charge have a greater ability to move within the membrane environment and to form clusters. The data in Fig. 6b support this insight, demonstrating that DPPE, which is uncharged and has no side chain in its hydrocarbon tail, has the highest NNC in the pure membrane, while AIPG, which possesses a charged head and two side chains in its hydrocarbon tail, exhibits the lowest NNC. Quantitative analysis of the NNC values reveals that the total absolute change in this parameter for 1MDP1 and 4MDP1 systems is 0.56 and 0.81, respectively. Therefore, the overall ability of 4MDP1 to change NNC is about 1.5 times that of 1MDP1.

It is important to note that the two lipids PAIPG and MAIPG, despite their great similarity in molecular structure and electrical charge, exhibit significantly different NNC values in the 1MDP1 system. This observation implies that additional factors, such as peptide–lipid and lipid–lipid interactions, play a role in influencing NNC values. Fig. 6b reveals that, for over half of the lipids, the changes in NNC within the 4MDP1 and 1MDP1 systems are comparable in magnitude and direction. However, certain lipids exhibit notable discrepancies in NNC changes between these systems. For instance, while the NNC values for PAIPE and DPPE in the 1MDP1 system closely resemble those of the pure membrane, they undergo significant reductions in the 4MDP1 system. Conversely, for DPPG, both systems display substantial and parallel changes in NNC, with the effects being markedly more pronounced in the 4MDP1 system.

#### 3.3.2 Radial distribution function

Another result that can be obtained from MD calculations is the Radial Distribution Function (RDF) parameter, which can provide valuable information about the arrangement of lipids in the membrane and the arrangement of water molecules in the phosphate headgroup.^69^ By comparing the RDF of the membrane in its pure state and in the presence of a drug, meaningful analysis regarding drug–membrane interactions can be conducted. Factors that promote the close arrangement of phospholipids of the same type contribute to an increase in RDF values. This phenomenon is influenced by several factors, including the fluidity of the system, the electrical charge assigned to individual lipids, and the energy of drug–lipid interactions.^69^ Enhanced system fluidity allows lipids to migrate more freely within the membrane, whereas electrostatic repulsions and strong drug–lipid interactions constrain the lateral mobility of lipids, thereby affecting the RDF.

**Fig. 7:**
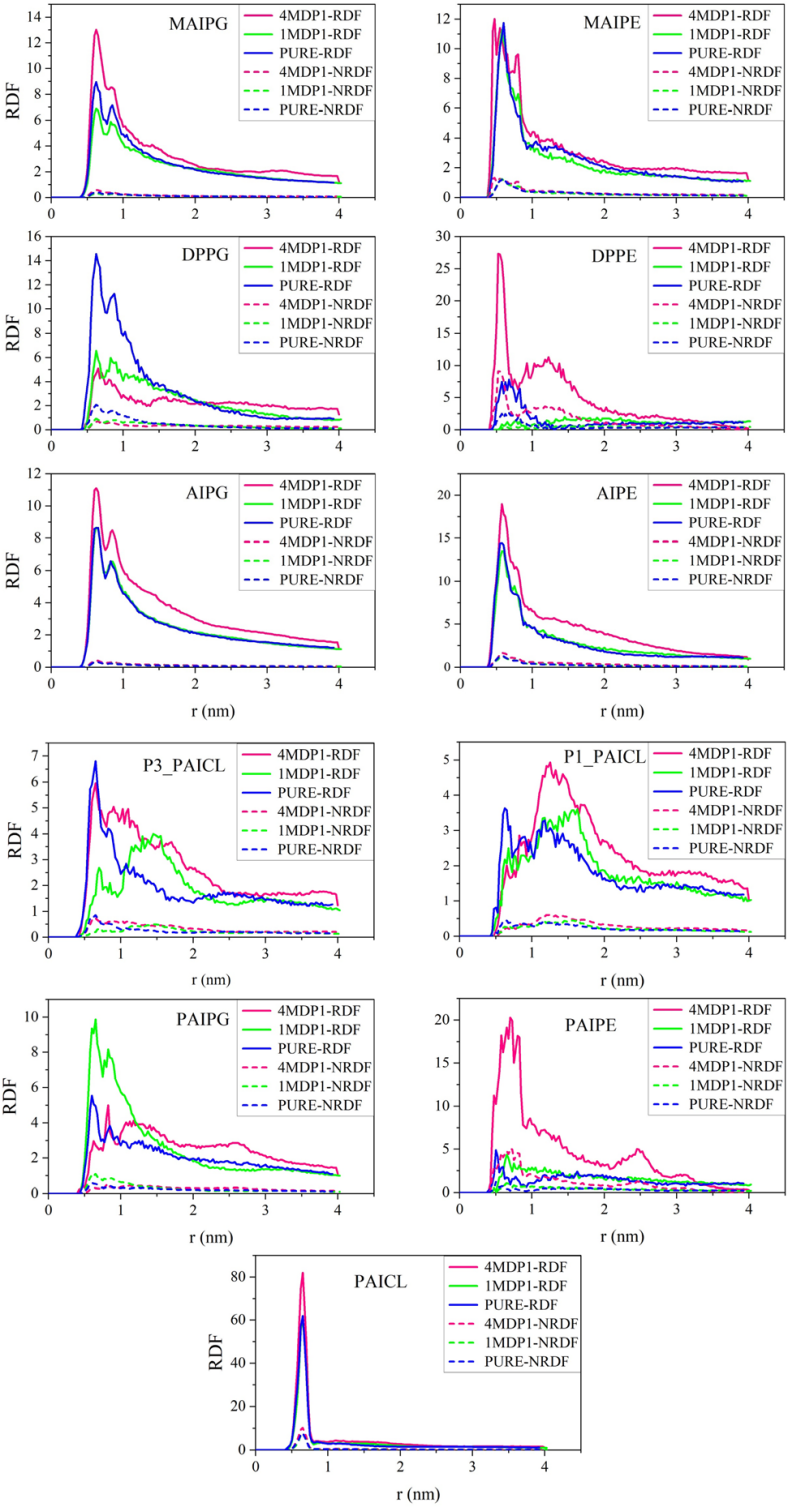
Radial distribution function plots along with the normalized radial distribution function (NRDF) for the 9 phospholipids in pure membranes and in the presence of 1MDP1 and 4MDP1.

RDF is anticipated to exhibit a robust correlation with NNC. However, it is important to note that while factors such as increased membrane fluidity tend to enhance both RDF and NNC, there are instances where changes in these parameters may diverge. This inconsistency arises because RDF analysis evaluates the likelihood of particles occupying different solvation layers, whereas NNC focuses solely on the probability of two particles being adjacent to one another. As a result, it is conceivable that the system might reduce the probability of forming solvation layers by promoting the formation of small, distinct clusters. Fig. 7 presents the RDF plots, along with the normalized radial distribution function (NRDF), calculated by dividing the RDF by the number of lipids of each type within the membrane. As depicted in the figure, no significant changes are observed in the RDF or NRDF for the 1MDP1 and 4MDP1 systems compared to the pure membrane, rendering the results for most phospholipids not analyzable. However, notable changes are evident in the RDF plots for three specific lipids: PAICL, DPPE, and PAIPE, particularly in the 4MDP1 system.

As shown in Fig. 7, the highest RDF value is observed for PAICL in the pure membrane, which is expected. PAICL has two adjacent phosphate groups in its molecular structure, inherently increasing the radial probability for each group relative to the other. The sharpness of the RDF peak, indicating a small displacement of the groups relative to each other, confirms this. Next, in the pure membrane, the highest RDF is from the peptides DPPE and DPPG. This is understandable considering that both of these lipids lack side chain in their hydrocarbon tails and are able to move more freely than other lipids in the membrane. According to Fig. 6, they also have the highest NNC value.

It is worth noting that if Fig. 6 were plotted based on just number of clusters (NC), the two phospholipids DPPE and PAIPE would exhibit the lowest values, producing results entirely contrary to those derived from RDF. This highlights the importance of considering statistical contributions and normalizing the properties by the number of phospholipid molecules of each type in the current study-a decision that demonstrates robust methodological grounding.

One of the points that can be realized from comparing Fig. 6 and 7 is that for phospholipids DPPE, PAIPE, and DPPG, the NNC for the 4MDP1 system has changed drastically compared to the pure membrane and the 1MDP1 system. The RDF has also undergone a significant change, and the increase in NNC is reciprocal to the decrease in RDF and *vice versa*. As mentioned above, this effect can be due to the fact that with increasing NNC, the probability of particles being present in the solvation layers of a hypothetical central particle decreases.

### 3.4 Ordered/disordered of carbon chains in selected phospholipids

The lipid membrane is an ordered liquid crystalline phase.^70^ Temperature, orientation across the membrane, and the length of the lipid tails are essential factors in understanding the dynamics and structure of the lipid bilayer membrane, which are related to the CH order parameter. The CH order parameter explains properties such as membrane strength, compressibility, and surface expansion modulus.^66^ Since the decrease or increase in membrane order due to the presence of an external agent, compared to the pure system, is considered both disorder in the membrane and a form of toxicity, this study uses the CH order parameter to analyze the effect of the MDP1 peptide on the bacterial membrane. The CH order parameter is calculated using the following formula:^71^

2 *S*_CD_ = ∣〈〈(3 cos^2^*θ* − 1)/2〉∣

where *θ* is the angle between a CD bond (in experimental) or a CH bond (in simulation) and the bilayer normal axis (*Z*), which is the normal axis of the membrane lipids.

**Fig. 8:**
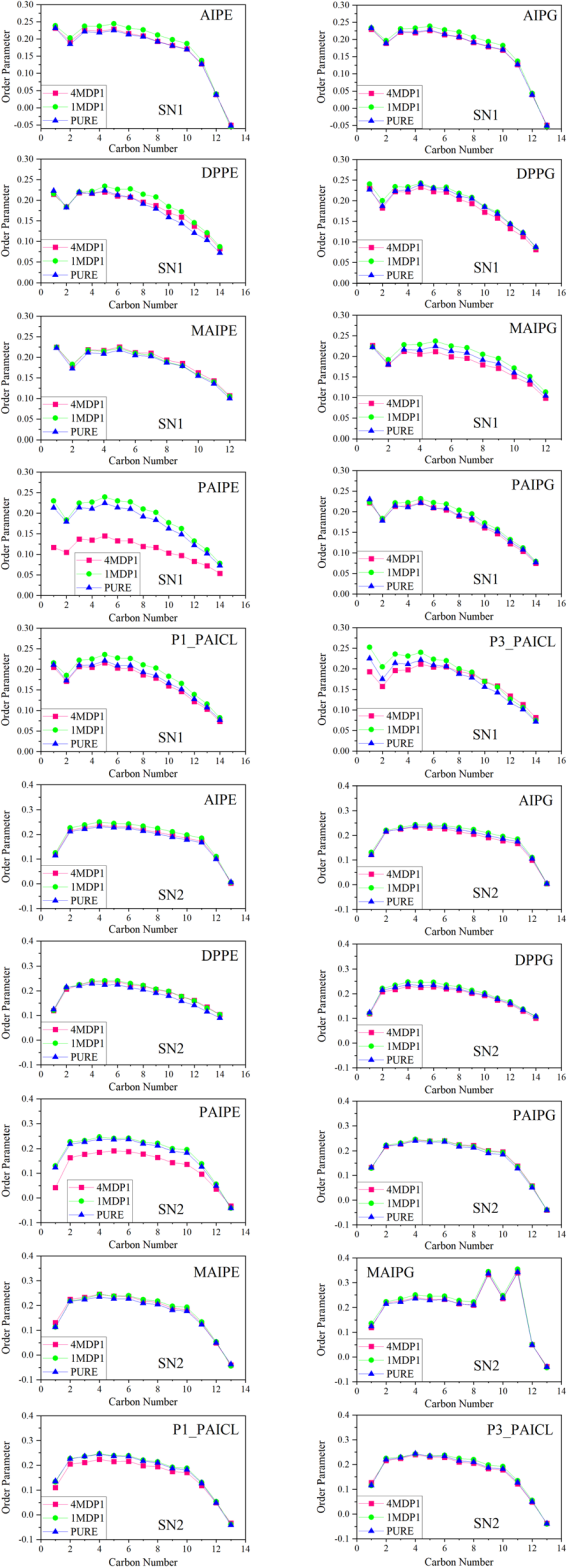
CH order parameters for SN1 and SN2 chains of phospholipids in all simulated systems. All results were obtained from the 600–1200 ns interval.

The alterations in the CH order parameter for both 1MDP1 and 4MDP1 systems are depicted in Fig. 8. To achieve a more precise assessment of the effect of the CH order parameter, the absolute changes in this parameter for the carbon atoms of the SN1 and SN2 chains of each phospholipid in the 1MDP1 and 4MDP1 systems, relative to the pure system, have been calculated and presented in Table S4. Analysis of Fig. 8 and Table S4 clearly reveals apparent changes in CH order for the SN1 chain, and comparatively smaller changes for the SN2 chain, indicating that the drug affected the membrane environment at both concentrations. Notably, the variations in CH order for both chains were more noticed in the 4MDP1 system than in the 1MDP1 system. The results presented in Table S4 reveal that, within the 1MDP1 system, the most significant absolute changes in the CH order parameter, approximately 0.4, are attributed to the lipids DPPE and AIPE. In contrast, the 4MDP1 system highlights lipid PAIPE as the primary contributor, with a value of 1.47, suggesting clustering effects. Notably, in the 4MDP1 system, PAIPE exerts the most substantial influence on both clustering variations and the CH order parameter. Although it may be challenging to directly correlate these observations with the molecular structures of the phospholipids, it is evident that uncharged lipids play the most prominent role in contributing to absolute changes in the CH order parameter across both systems.

Conversely, variations in the CH order parameter appear to be primarily governed by two distinct properties of the lipid hydrocarbon chains. The first factor influencing the change in the CH order parameter is the length of the hydrocarbon chain. As the chain length increases, the lipid molecule undergoes more substantial structural adjustments to achieve stability within the membrane, resulting in a greater change in the order parameter. The second factor is the presence of a side chain in the hydrocarbon tail, which similarly demands significant structural modifications for the molecule to attain thermodynamic stability in the membrane. Consequently, it can be concluded that uncharged lipid molecules possessing either a long hydrocarbon tail or a side chain are likely to induce the largest absolute changes in the CH order parameter. Remarkably, all three of these characteristics coexist in the PAIPE phospholipid.

### 3.5 Phospholipids movement

To elucidate the influence of the cationic amphipathic peptide MDP1 on the time-resolved interactions of the bacterial membrane lipids, the computation of the mean square displacement (MSD) of the membrane phospholipids on the outer leaflet (exposed to the peptide) during a 1200 ns molecular dynamics simulation. This analysis provides a quantitative understanding of how peptide loading modulates the lateral mobility of membrane lipids. The lateral diffusion coefficient (*D*_L_) can be calculated using the formula:^72^3
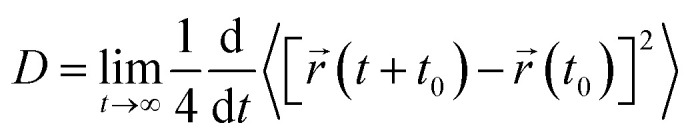
where *D* is the diffusion coefficient, *t* is the time, and 〈⋯〉 represents the time average of the lipid motion time series, *r*(*t*).

**Fig. 9:**
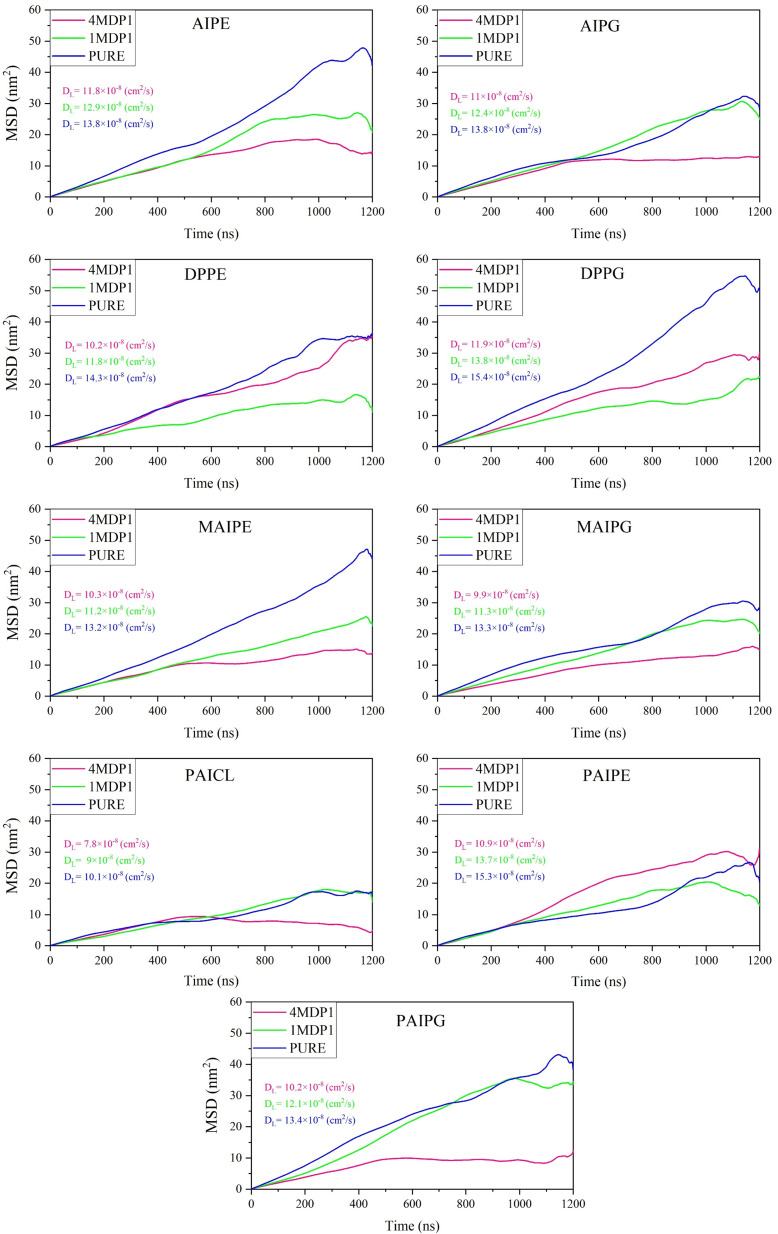
MSD of outer leaflet phospholipids in the simulated systems. Accompanied by the corresponding *D*_L_ of each lipid in the same color.

As seen in Fig. 9, the MSD decreased across most lipid species in the presence of MDP1, with the strongest suppression observed in the 4MDP1 system.

This trend indicates a dose-dependent reduction in lipid mobility, consistent with previous reports demonstrating that AMPs reduce lipid diffusion by enhancing local ordering or promoting peptide–lipid clustering at the membrane interface.^73^ Notably, since only outer leaflet lipids were analyzed, the observed changes represent localized perturbations associated with direct peptide contact rather than global bilayer effects, a phenomenon widely documented in AMP–membrane systems.^74^

#### 3.5.1 PE-containing lipids (AIPE, MAIPE, DPPE, PAIPE)

The presence of MDP1 led to a monotonic decrease in MSD compared with the pure bilayer. Both 1MDP1 and 4MDP1 systems exhibited progressively lower diffusion rates, indicating a gradual lateral confinement of PE headgroups as peptide concentration increased. This suggests enhanced peptide–lipid coupling and possible stabilization of locally ordered domains around peptide adsorption sites. Such behavior aligns with earlier findings that amphipathic α-helical peptides locally rigidify PE-rich bilayers and suppress lateral lipid diffusion.^75^

In contrast, DPPE displayed a nonlinear response: MSD decreased in 1MDP1 but partially recovered in 4MDP1, approaching the pure bilayer value. This could reflect heterogeneous peptide–lipid reorganization, where local compensatory mechanisms such as lateral lipid redistribution or microdomain segregation restore partial fluidity at higher peptide coverage. A similar non-monotonic dependence of diffusion on peptide/lipid ratio has been observed for other membrane-active peptides.^76^

Interestingly, in the case of PAIPE, the highest peptide concentration resulted in a noticeable increase in MSD relative to the pure bilayer, whereas the presence of one peptide caused reduced mobility. This opposite trend suggests that at high peptide surface densities, membrane softening or local thinning may occur, enhancing lipid diffusion in regions with higher curvature or disorder.

#### 3.5.2 PG-containing lipids (AIPG, DPPG, MAIPG, PAIPG)

For PG-containing lipids, which have negatively charged headgroups, MDP1 was found to consistently inhibit lipid movement. In AIPG, a single peptide caused a slight increase in MSD compared to the pure system, possibly due to localized disorder at low peptide density. However, the four-peptide system exhibited a pronounced decrease, reflecting strong electrostatic binding and the formation of peptide–PG complexes (see Fig. 2).

Similarly, the MSD values of DPPG, MAIPG and PAIPG lipids decreased progressively as the peptide concentration increased. This behavior is characteristic of the electrostatically driven immobilization of anionic lipids, whereby cationic residues (particularly lysine and arginine clusters) interact with the negatively charged phosphate and glycerol groups. This reduces the lateral diffusion of lipids.

This process has been corroborated by calorimetric and spectroscopic studies which report increased phase transition temperatures and reduced headgroup hydration in anionic membranes exposed to cationic peptides.^77^ Furthermore, charge-driven lipid clustering has been widely reported in models of peptide–membrane interactions.^78^

#### 3.5.3 CL-containing lipid (PAICL)

The behavior of PAICL was rather unique compared to other lipids. In the case of the 1MDP1 system, its MSD remained comparable to that of the pure bilayer, while in the 4MDP1 system there was a considerable decrease in lipid mobility. PAICL has a doubly negatively charged, inverted-coned geometry, and thus, is expected to have strong electrostatic interactions with cationic peptides, as seen in Fig. 2 This could explain the formation of lipid-PAICL regions and the localized restriction of lipid mobility around sites of peptide adsorption. These observations coincide with prior studies which pointed out that cardiolipins enhance the rigidity of the membrane and can also inhibit the mechanistic disruptions that peptides induce, thereby modulating the overall response of the bilayer to AMP binding.^79,80^

Therefore, it can be observed that by adding peptide to the membrane, the system becomes more rigid. With increasing peptide concentration, viscosity increases and fluidity decrease. Additionally, the position of each of the nine membrane phospholipids undergoes significant changes.

### 3.6 Peptide anisotropy

The proximity of peptides to each other and to the membrane can lead to their self-assembly.^6^ The radius of gyration provides information about this change in arrangement, the structural features of the peptide, and the distribution of possible structures.^81^ Eigenvalues of the rotational tensor functions *λ*_1_, *λ*_2_, and *λ*_3_ are such that *λ*_1_ ≥ *λ*_2_ ≥ *λ*_3_, and the square of the radius of gyration equals their sum: *R*_g_^2^ = *λ*_1_ + *λ*_2_ + *λ*_3_. To investigate the structural changes of peptides, the relative shape anisotropy parameter (RSA) obtained from the radius of gyration is used. RSA, which reveals the symmetry and dimensions of the structure in polymers, is obtained using the following equation:^82^4
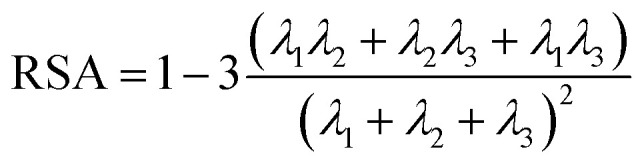


**Fig. 10:**
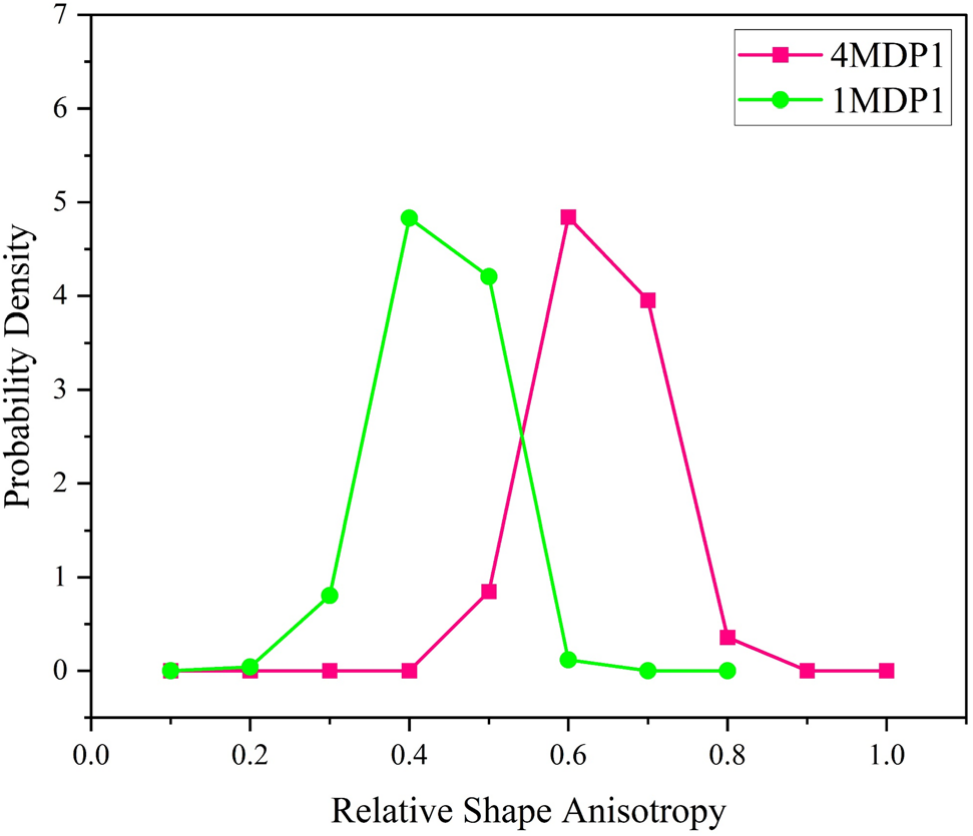
The relative shape anisotropy parameter of MDP1 molecules for 4MDP1 and 1MDP1systems. All results were obtained from the 600–1200 ns interval.

The RSA parameter ranges between zero and one, with values near one representing a linear chain and values near zero corresponding to a completely symmetrical structure. For a symmetrical planar structure, the RSA is approximately 0.25. Fig. 10 depicts the RSA probability density values, highlighting differences between the 1MDP1 and 4MDP1 systems. In the 1MDP1 system, the highest probability density spans from 0.2 to 0.6, peaking at 0.4, indicating that the peptide predominantly adopts a spherical shape when interacting with the membrane. In contrast, the 4MDP1 system shows RSA probability densities ranging from 0.4 to 0.8, with a peak at 0.6, suggesting that peptides are more inclined to form linear and cylindrical structures compared to those in the 1MDP1 system.

### 3.7 Secondary structure

The secondary structure of the peptide and its stability during peptide–membrane interactions influence its therapeutic qualities.^7,9^ Studies indicate that the peptide's secondary structure affects lipid–membrane interactions and self-aggregation.^83,84^ This study employed the DSSP program to ascertain the parameters of secondary structure.^85,86^Table 3 presents the secondary structure parameters of the peptides for the 1MDP1 and 4MDP1 systems. According to the data presented in this table, the average value of all secondary structure parameters in the 4MDP1 system differ notably from those in the 1MDP1 system, with the most substantial variation observed in the turn parameter. Additionally, the α-helix structure in the 4MDP1 system is, on average, about 4% higher than in the 1MDP1 system, suggesting that 4MDP1 may be more structured than 1MDP1. Fig. S7 also shows the temporal changes in the secondary structure of the peptide. As depicted in this figure, glycine acts as a helix-destroying factor, and for all peptides, the dominant structure is α-helix. Furthermore, the time evaluations of replicas are illustrated in Fig. S8 and S9. Additionally, in the 1MDP1 system, the intermediate structure of the peptide in the glycine-12 region coils up to 500 ns and then transitions to α-helix. In the 4MDP1 system, most deviations from the helix structure are observed at the beginning and end of the peptide. Furthermore, a 5-helix and turn structure is formed in the middle of the peptide.

**Table 3:** The averaged population of the secondary structure content from DSSP analysis^a^

Simulated systems	Structures	Coil (%)	Bend (%)	Turn (%)	α-Helix (%)	5-Helix (%)	3-Helix (%)
	Number of peptide						
1 MDP1	MDP1	26.01 (±0.05)	4.61 (±0.25)	2.47 (±0.85)	63.45 (±0.06)	3.37 (±0.95)	0.09 (±5.15)
4 MDP1	MDP1_1	26.7 (±1.79)	5.89 (±0.76)	7 (±1.63)	55.2 (±2.39)	—	5.20 (±1.21)
	MDP1_2	15.99 (±0.59)	1.81 (±0.74)	4.55 (±0.79)	74.14 (±3.21)	3.51 (±3.02)	0.01 (±0.00)
	MDP1_3	20.08 (±0.91)	5.19 (±0.38)	3.05 (±0.83)	71.57 (±0.40)	0.04 (±0.03)	0.06 (±0.04)
	MDP1_4	25.15 (±0.87)	2.05 (±0.35)	7.57 (±0.31)	64.58 (±0.98)	0.05 (±0.02)	0.62 (±0.07)

a All results were obtained from the 600–1200 ns interval.

## 4. Conclusion

In this study, we examined the interaction of two different concentrations of MDP1—1MDP1 and 4MDP1—with a bacterial cell membrane composed of nine distinct phospholipid types using MD simulations. The findings indicate that increasing the concentration of MDP1 induces self-aggregation and enhances the contribution of electrostatic interactions in drug–membrane interactions. Specifically, with a 4-fold increase in the number of MDP1, the contribution of electrostatic interactions became 3.19 times that of the 1MDP1 system. While, the contribution of vdW interactions only underwent a slight change due to self-aggregation. Thus, this research findings indicate that incorporating factors that strengthen electrostatic interactions may enhance the efficacy of MDP1. Furthermore, in both the 1MDP1 and 4MDP1 systems, the PG-type phospholipids, due to their distinct molecular structure, plays a pivotal role in reinforcing electrostatic and vdW interactions, as well as facilitating hydrogen bond formation.

The results of the present study, in agreement with previous findings, suggest that the amino acid arginine plays a significant role not only in membrane–drug binding energy but also in the formation of hydrogen bonds for both 1MDP1 and 4MDP1. Moreover, results indicate that in both systems, 1MDP1 and 4MDP1, the membrane becomes more rigid and viscous with the addition of the peptide. Based on the study's findings, an uncharged phospholipid molecule like PAIPE with a long hydrocarbon tail or side chain can cause the most significant change in the CH order parameter.

Furthermore, it was observed that the presence of the drug does not significantly impact membrane thickness. On the other hand, electron density calculations indicate that the negative electrical charges of the phosphate head group of lipids are neutralized by interacting with the positive charges of peptides. This reduces the tendency of both sodium ions and water molecules to migrate to this region in the 4MDP1 system compared to the pure and 1MDP1 systems. Finally, the RDF calculation results align with the RNC results, demonstrating that the presence of 4MDP1 has the most significant impact on the RDF of the two phospholipids DPPE and PAIPE.

## Conflicts of interest

There are no conflicts to declare.

## Supplementary Material

RA-016-D5RA06511A-s001

RA-016-D5RA06511A-s002

RA-016-D5RA06511A-s003

## Data Availability

All of the initial inputs and the data supporting this article have been included as part of the supplementary information (SI). Supplementary information is available. See DOI: https://doi.org/10.1039/d5ra06511a.

## References

[cit1] Sizar O. , Leslie S. W. and Unakal C. G., Gram-Positive Bacteria, in StatPearls, Treasure Island StatPearls, FL, 2025, PMID: 29261915 29261915

[cit2] Doernberg S. B., Lodise T. P., Thaden J. T., Munita J. M., Cosgrove S. E., Arias C. A., Boucher H. W., Corey G. R., Lowy F. D., Murray B., Miller L. G., Holland T. L. (2017). Clin. Infect. Dis..

[cit3] Carcione D., Intra J., Andriani L., Campanile F., Gona F., Carletti S., Mancini N., Brigante G., Cattaneo D., Baldelli S., Chisari M., Piccirilli A., Di Bella S., Principe L. (2023). Pharmaceuticals.

[cit4] Joodaki F., Martin L. M., Greenfield M. L. (2022). Langmuir.

[cit5] Zasloff M. , in Advances in Experimental Medicine and Biology, Springer New York LLC, 2019, vol. 1117, pp. 3–6 10.1007/978-981-13-3588-4_1 30980349

[cit6] Maleš M., Zoranić L. (2022). Membranes.

[cit7] Mirnejad R., Fasihi-Ramandi M., Behmard E., Najafi A., Moosazadeh Moghaddam M. (2023). Chem. Pap..

[cit8] Corrêa J. A. F., Evangelista A. G., Nazareth T. D. M., Luciano F. B. (2019). Materialia.

[cit9] Ma R., Wong S. W., Ge L., Shaw C., Siu S. W. I., Kwok H. F. (2020). Mol. Ther. Oncolytics.

[cit10] Ji S., An F., Zhang T., Lou M., Guo J., Liu K., Zhu Y., Wu J., Wu R. (2024). Eur. J. Med. Chem..

[cit11] Elyass M. E., Mahdi A. A., Semeih A. E., Eltaib F. I., Attitalla I. H. (2021). Microb. Pathog..

[cit12] Quemé-peña M., Juhász T., Kohut G., Ricci M., Singh P., Szigyártó I. C., Papp Z. I., Fülöp L., Beke-somfai T. (2021). Int. J. Mol. Sci..

[cit13] Herrera-León C., Ramos-Martín F., Antonietti V., Sonnet P., D'Amelio N. (2022). FEBS J..

[cit14] Lee J., Jung S. W., Cho A. E. (2016). Langmuir.

[cit15] Jahangiri S., Jafari M., Arjomand M., Mehrnejad F. (2018). J. Cell. Biochem..

[cit16] Mishra B., Golla R. M., Lau K., Lushnikova T., Wang G. (2016). ACS Med. Chem. Lett..

[cit17] Wang G., Hanke M. L., Mishra B., Lushnikova T., Heim C. E., Chittezham Thomas V., Kielian T. (2014). ACS Chem. Biol..

[cit18] Khavani M., Mehranfar A., Mofrad M. R. K. (2025). J. Biomol. Struct. Dyn..

[cit19] Thankappan B., Thomas A., Sakthivadivel A., Shanmuganathan N., Angayarkanni J. (2023). Colloids Surf., B.

[cit20] Sabapathy T., Deplazes E., Mancera R. L. (2020). Int. J. Mol. Sci..

[cit21] Miyazaki Y., Shinoda W. (2022). Biochim. Biophys. Acta Biomembr..

[cit22] Sun L., Wang S., Tian F., Zhu H., Dai L. (2022). Biophys. J..

[cit23] Memariani H., Memariani M., Shahidi-Dadras M., Nasiri S., Akhavan M. M., Moravvej H. (2019). Appl. Microbiol. Biotechnol..

[cit24] Akbari R., Hakemi Vala M., Hashemi A., Aghazadeh H., Sabatier J. M., Pooshang Bagheri K. (2018). Amino Acids.

[cit25] Akbari R., Hakemi Vala M., Sabatier J. M., Pooshang Bagheri K. (2022). Amino Acids.

[cit26] Pathak R. K. , Singh D. B., Singh S., Swati S. and Kumar Keshvarani R., Recent Advances in Computer Aided Drug Designing, Nova Science, 2021

[cit27] Ouyang J., Sheng Y., Wang W. (2022). Cells.

[cit28] Paro S. and Imler J. L., Immunity in Insects, 2016, vol. 1

[cit29] Verma A. and Ogata S., Computational Modelling of Deformation and Failure of Bone at Molecular Scale, 2022, vol. 99

[cit30] Akbari R., Hakemi Vala M., Hashemi A., Aghazadeh H., Sabatier J. M., Pooshang Bagheri K. (2018). Amino Acids.

[cit31] Dashtbin S., Razavi S., Ganjali Koli M., Barneh F., Ekhtiari-Sadegh S., Akbari R., Irajian G., Pooshang Bagheri K. (2024). Front. Microbiol..

[cit32] Ekhtiari-Sadegh S., Samani S., Barneh F., Dashtbin S., Shokrgozar M. A., Pooshang Bagheri K. (2024). Front. Bioeng. Biotechnol..

[cit33] Eisenberg D. , Gribskov M., and Terwilliger T. C., Melittin (PDB ID: 2MLT), RCSB Protein Data Bank, 1990, Available from: https://www.rcsb.org/structure/2MLT.

[cit34] Sali A. (1996). Acta Crystallogr., Sect. A.

[cit35] Bordoli L., Kiefer F., Arnold K., Benkert P., Battey J., Schwede T. (2009). Nat. Protoc..

[cit36] Pogozheva I. D., Armstrong G. A., Kong L., Hartnagel T. J., Carpino C. A., Gee S. E., Picarello D. M., Rubin A. S., Lee J., Park S., Lomize A. L., Im W. (2022). J. Chem. Inf. Model..

[cit37] Jorgensen W. L., Chandrasekhar J., Madura J. D., Impey R. W., Klein M. L. (1983). J. Chem. Phys..

[cit38] Ginter T., Dujardin A., Roumans S., Rothschild L. J., Rheinstädter M. C. (2024). Int. J. Astrobiol..

[cit39] Lee J., Patel D. S., Ståhle J., Park S. J., Kern N. R., Kim S., Lee J., Cheng X., Valvano M. A., Holst O., Knirel Y. A., Qi Y., Jo S., Klauda J. B., Widmalm G., Im W. (2019). J. Chem. Theory Comput..

[cit40] Wu E. L., Cheng X., Jo S., Rui H., Song K. C., Dávila-Contreras E. M., Qi Y., Lee J., Monje-Galvan V., Venable R. M., Klauda J. B., Im W. (2014). J. Comput. Chem..

[cit41] Brown T. P., Santa D. E., Berger B. A., Kong L., Wittenberg N. J., Im W. (2024). Curr. Opin. Struct. Biol..

[cit42] Park S., Choi Y. K., Kim S., Lee J., Im W. (2021). J. Chem. Inf. Model..

[cit43] Feng S., Park S., Choi Y. K., Im W. (2023). J. Chem. Theory Comput..

[cit44] Jo S., Lim J. B., Klauda J. B., Im W. (2009). Biophys. J..

[cit45] Jo S., Kim T., Im W. (2007). PLoS One.

[cit46] Gee S., Glover K. J., Wittenberg N. J., Im W. (2024). ChemPlusChem.

[cit47] Best R. B. , Zhu X., Shim J., Lopes P. E. M., Mittal J., Feig M. and Mackerell A. D.

[cit48] Klauda J. B., Venable R. M., Freites J. A., O'Connor J. W., Tobias D. J., Mondragon-Ramirez C., Vorobyov I., MacKerell A. D., Pastor R. W. (2010). J. Phys. Chem. B.

[cit49] Abraham M. J., Murtola T., Schulz R., Páll S., Smith J. C., Hess B., Lindah E. (2015). SoftwareX.

[cit50] Bauer P. , Hess B. and Lindahl E., GROMACS 2022.2 Manual (2022.2), 2022, Zenodo, 10.5281/zenodo.6637572

[cit51] Bauer P. , Hess B. and Lindahl E., GROMACS 2022, Source code (Version 2022), 2022, Zenodo, 10.5281/zenodo.6103835

[cit52] Evans D. J., Holian B. L. (1985). J. Chem. Phys..

[cit53] Parrinello M., Rahman A. (1981). J. Appl. Phys..

[cit54] Hess B., Bekker H., Berendsen H. J. C., Fraaije J. G. E. M. (1997). J. Comput. Chem..

[cit55] Hockney R. W., Goel S. P., Eastwood J. W. (1974). J. Comput. Phys..

[cit56] Darden T., York D., Pedersen L. (1993). J. Chem. Phys..

[cit57] Corey R. A., Vickery O. N., Sansom M. S. P., Stansfeld P. J. (2019). J. Chem. Theory Comput..

[cit58] Fernández J. A. (2020). J. Indian Inst. Sci..

[cit59] Zhu X., Lopes P. E. M., MacKerell Jr A. D. (2012). Wiley Interdiscip. Rev. Comput. Mol. Sci..

[cit60] Vanommeslaeghe K., Hatcher E., Acharya C., Kundu S., Zhong S., Shim J., Darian E., Guvench O., Lopes P., Vorobyov I. (2010). J. Comput. Chem..

[cit61] Tsuchiya H., Mizogami M. (2013). Anesthesiol. Res. Pract..

[cit62] Via M. A., Klug J., Wilke N., Mayorga L. S., Del Pópolo M. G. (2018). Phys. Chem. Chem. Phys..

[cit63] Ding W., Palaiokostas M., Wang W., Orsi M. (2015). J. Phys. Chem. B.

[cit64] Catalina-Hernandez E., Aguilella-Arzo M., Peralvarez-Marin A., Lopez-Martin M. (2025). J. Chem. Inf. Model..

[cit65] Li T., Wang Z., Guo J., de la Fuente-Nunez C., Wang J., Han B., Tao H., Liu J., Wang X. (2023). Sci. Total Environ..

[cit66] Moradi S., Nowroozi A., Shahlaei M. (2019). RSC Adv..

[cit67] Klähn M., Zacharias M. (2013). Phys. Chem. Chem. Phys..

[cit68] Ghosh S., Devanand T., Baul U., Vemparala S. (2019). J. Chem. Phys..

[cit69] Azizi K., Koli M. G. (2016). J. Mol. Graph. Model..

[cit70] Dzikovski B. and Freed J., in Encyclopedia of Biophysics, 2019, pp. 1–8

[cit71] Yu Y., Krämer A., Venable R. M., Brooks B. R., Klauda J. B., Pastor R. W. (2021). J. Chem. Theory Comput..

[cit72] Boroushaki T., Ganjali Koli M., Eshaghi Malekshah R., Dekamin M. G. (2023). RSC Adv..

[cit73] Interactions P. M., Smith-dupont K. B., Guo L., Gai F. (2010). Biochemistry.

[cit74] Epand R. M., Epand R. F. (2009). Biochim. Biophys. Acta Biomembr..

[cit75] Zemel A., Ben-Shaul A., May S. (2005). Eur. Biophys. J..

[cit76] Joanne P., Galanth C., Goasdoué N., Nicolas P., Sagan S., Lavielle S., Chassaing G., El C., Alves I. D. (2009). Biochim. Biophys. Acta Biomembr..

[cit77] Hädicke A., Blume A. (2017). Biochim. Biophys. Acta Biomembr..

[cit78] Paterson D. J., Tassieri M., Reboud J., Wilson R., Cooper J. M. (2017). Proc. Natl. Acad. Sci. U. S. A..

[cit79] Calderón-Rivera N., Múnera-Jaramillo J., Jaramillo-Berrio S., Suesca E., Manrique-Moreno M., Leidy C. (2023). Membranes.

[cit80] Rashid R., Veleba M., Kline K. A. (2016). Front. Cell Dev. Biol..

[cit81] Gholami R., Azizi K., Ganjali Koli M. (2024). Sci. Rep..

[cit82] Arkin H., Janke W. (2013). J. Chem. Phys..

[cit83] Aghazadeh H., Ganjali M., Reza K., Kamran R., Bagheri P. (2020). J. Comput. Aided Mol. Des..

[cit84] Silvestro L., Axelsen P. H. (2000). Biophys. J..

[cit85] Touw W. G., Baakman C., Black J. (2015). A series of PDB-related databanks for everyday needs. Nucleic Acids Res..

[cit86] Kabsch W., Sander C. (1983). Biopolymers.

