# Supplementary material for: Structural characterization and thermodynamic behavior of melittin-derived peptide interactions with gram-positive bacterial cell membranes using molecular dynamics simulation
Source: RSC Adv. 2026 Mar 2;16(13):11562–79. doi: 10.1039/d5ra06511a (PMC12951287; doi:10.1039/d5ra06511a)
Supplement: RA-016-D5RA06511A-s003 [file RA-016-D5RA06511A-s003.pdf]

Supplementary Information (SI) for RSC Advances.
This journal is © The Royal Society of Chemistry 2026

Supplementary Information for: Structural Characterization and Thermodynamic Behavior of Melittin-Derived Peptide Interactions with Gram-Positive Bacterial Cell Membranes Using Molecular Dynamics Simulation

Yosra Delshad[a], Khaled Azizi[a,b,], Federico Fogolari[c], Mokhtar Ganjali Koli[d]*

[a]*Department of Chemistry, University of Kurdistan, Sanandaj, Iran*

[b]*Computational Chemistry Laboratory, Kask Afrand Exire Ltd., Sanandaj, Iran*

[c]*Dipartimento di Scienze Matematiche Informatiche e Fisiche (DMIF), University of Udine, Via delle Scienze 206, 33100 Udine, Italy*

[d]*Department of Energy, Politecnico di Torino, Corso Duca degli Abruzzi 24, Torino 10129, Italy*

Contents

Figure S1: Molecular structure of different phospholipids used as components of gram-positive-bacteria membrane in this study……………………………………………………….……3

Figure S2: Initial and final configurations of the 1MDP1 *Replica_2* and *Replica_3* towards the gram-positive bacteria membrane……………………………………………………………..5

Figure S3: Initial and final configurations of the 4MDP1 *Replica_2* and *Replica_3* towards the gram-positive bacteria membrane……………………………………………………………..6

Table S1: The averaged population of the secondary structure content from DSSP analysis for each peptide in 1MDP1-Water system and 4MDP1-Water system………………………………….…7

Figure S4: Root mean square deviation and radius of gyration of each peptide in 4MDP1-water system as a function of time ……………………………………………………….........................8

Equilibration Procedures…………………………………………………………………9

Figure S5: Interaction energies between peptides and different phospholipids of the 1MDP1 *Replica_2* and *Replica_3 systems*…………………………………………………………....10

Figure S6: Interaction energies between peptides and different phospholipids of the 4MDP1 *Replica_2* and *Replica_3 systems*…………………………………………………………....11

Table S2: Net contribution (first row) and normalized contribution (second row) of each of the 9 phospholipids to the total electrostatic and vdW interaction energy, expressed as a percentage for the two systems 1MDP1 and 4MDP1………………….…………………………………….…12

Table S3: Total electrostatic and vdW interaction energy values of peptide residues for the 1MDP1 and 4MDP1 systems………………………………………………………..……..13

Table S4: The absolute changes of CH order parameter for the carbon atoms of the SN1 and SN2 chains of each phospholipid in the 1MDP1 and 4MDP1 systems, relative to the pure system……………………………………………………………………………………14

Figure S7: Secondary structures of MDP1 molecules (a) in 1 MDP1, (b) in 4 MDP1systems

……………………..………………………………………………….……………..…..15

Figure S8: Secondary structures of MDP1 molecules for 1MDP1 systems, *Replica_2* and *Replica_3*…….……………………….…………………………………………………16

Figure S9: Secondary structures of MDP1 molecules for 4MDP1 systems, *Replica_2* and *Replica_3*…….……………………….…………………………………………………18

Table S5: Contribution of each amino acid to electrostatic and van der Waals interactions with 1MDP1 and 4MDP1 system……………………………………………………………………..20

SN1

SN2

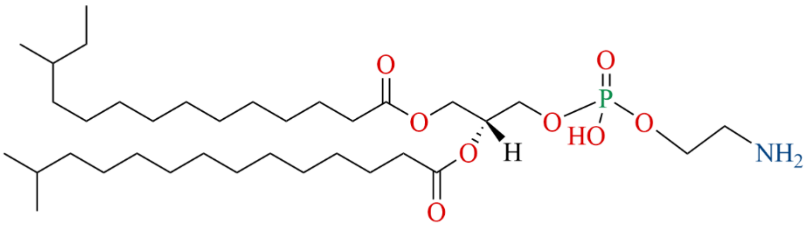

AIPE

SN1

SN2

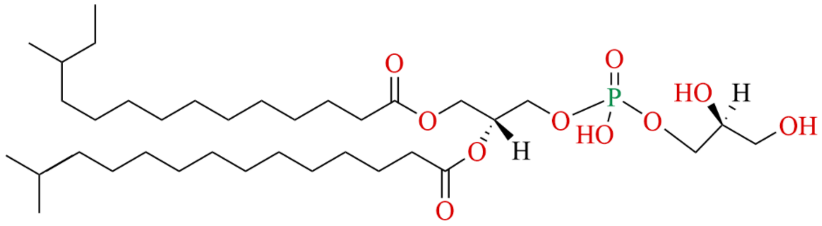

AIPG

SN1

SN2

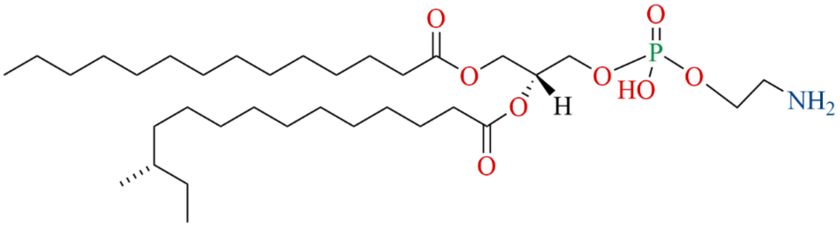

MAIPE

SN1

SN2

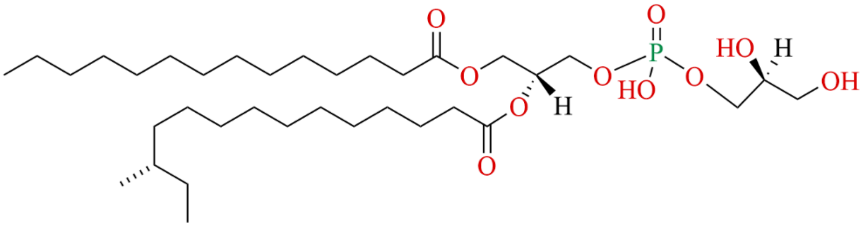

MAIPG

SN1

SN2

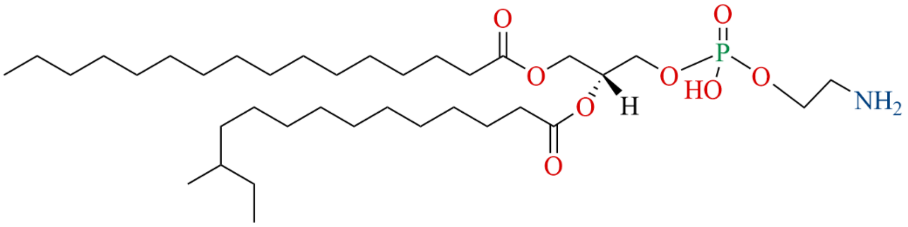

PAIPE

SN1

SN2

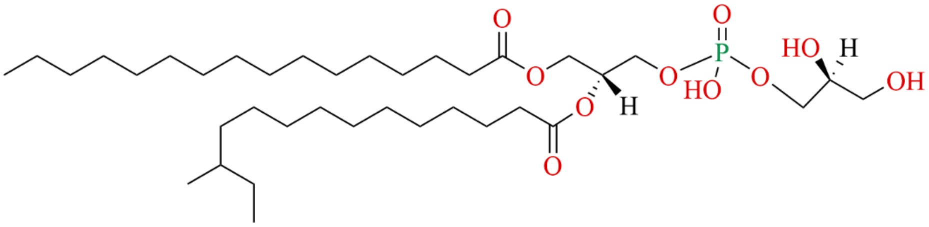

PAIPG

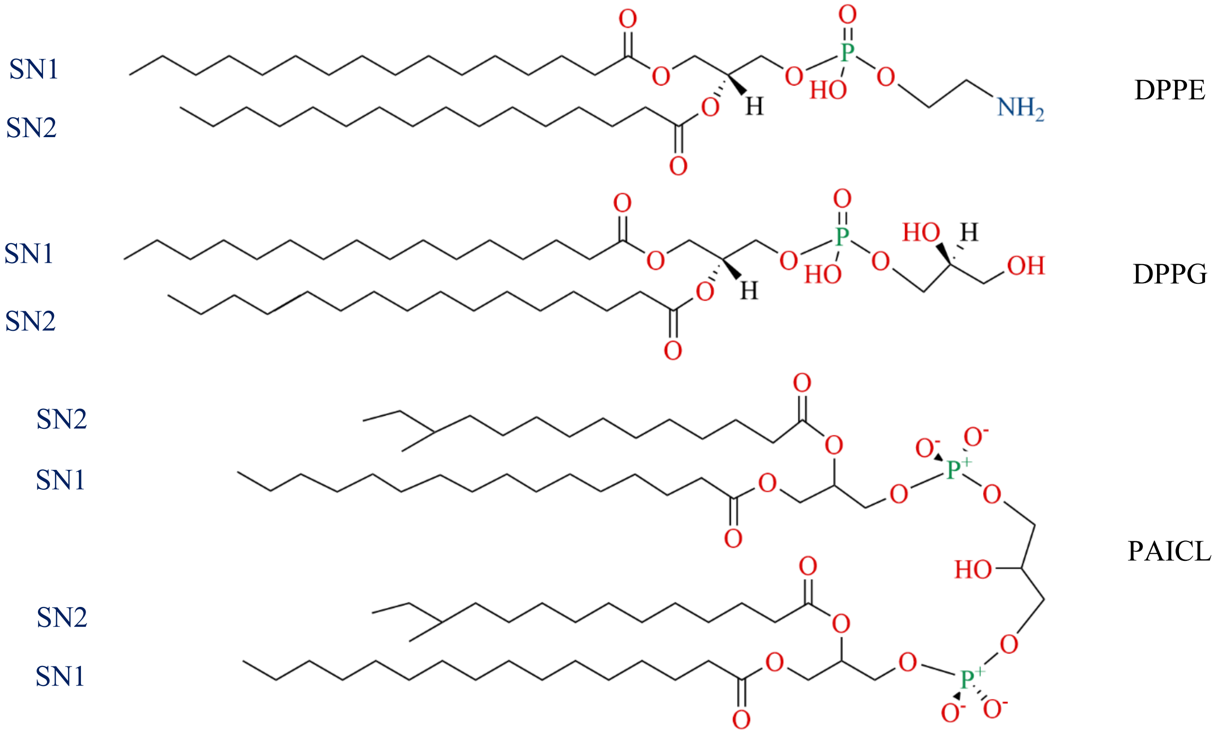

Figure S1: Molecular structure of different lipids used as components of gram-positive bacteria membrane in this study.

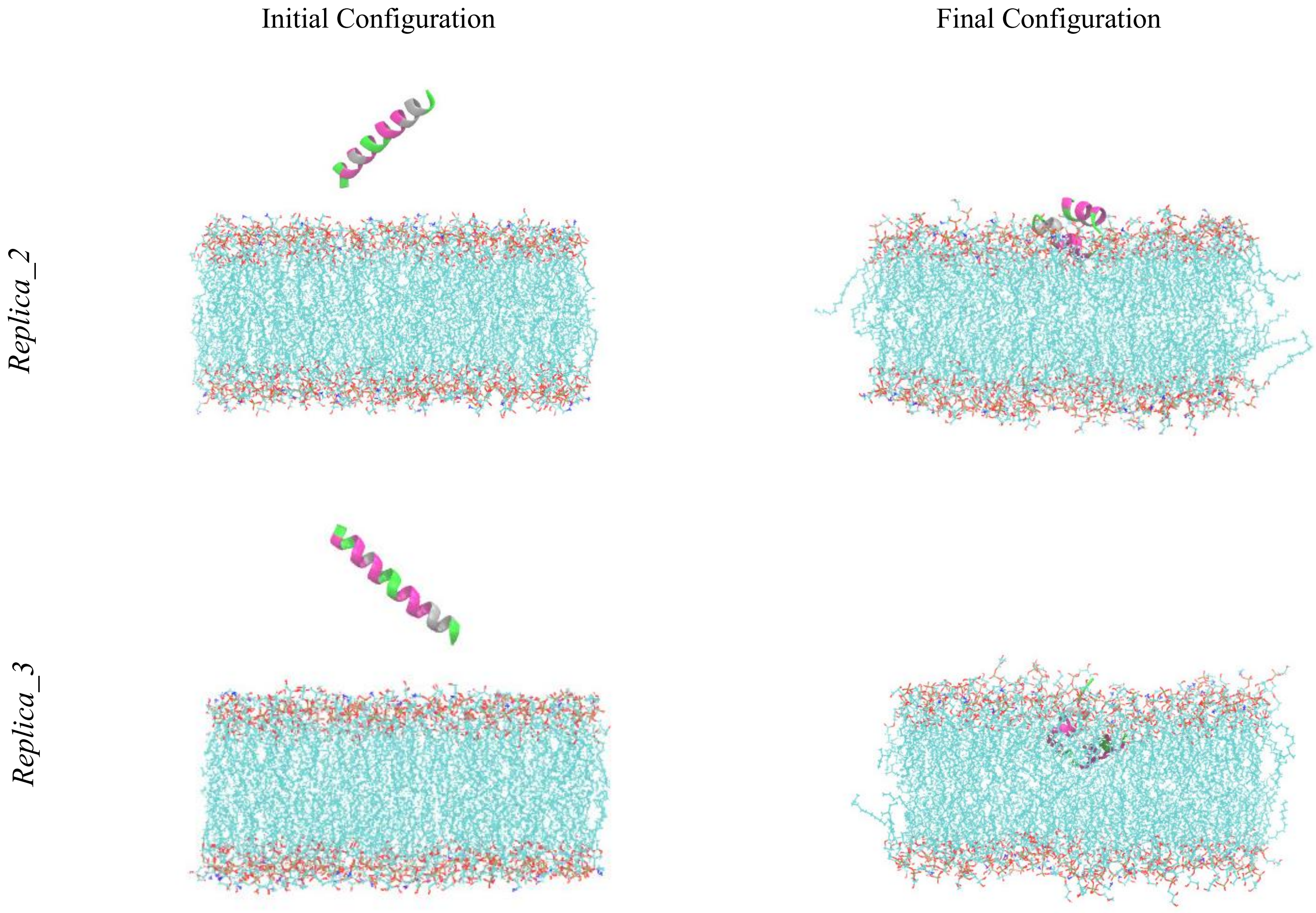

Figure S2: Initial and final configurations for *Replica_2* and *Replica_3* for 1MDP1 systems.

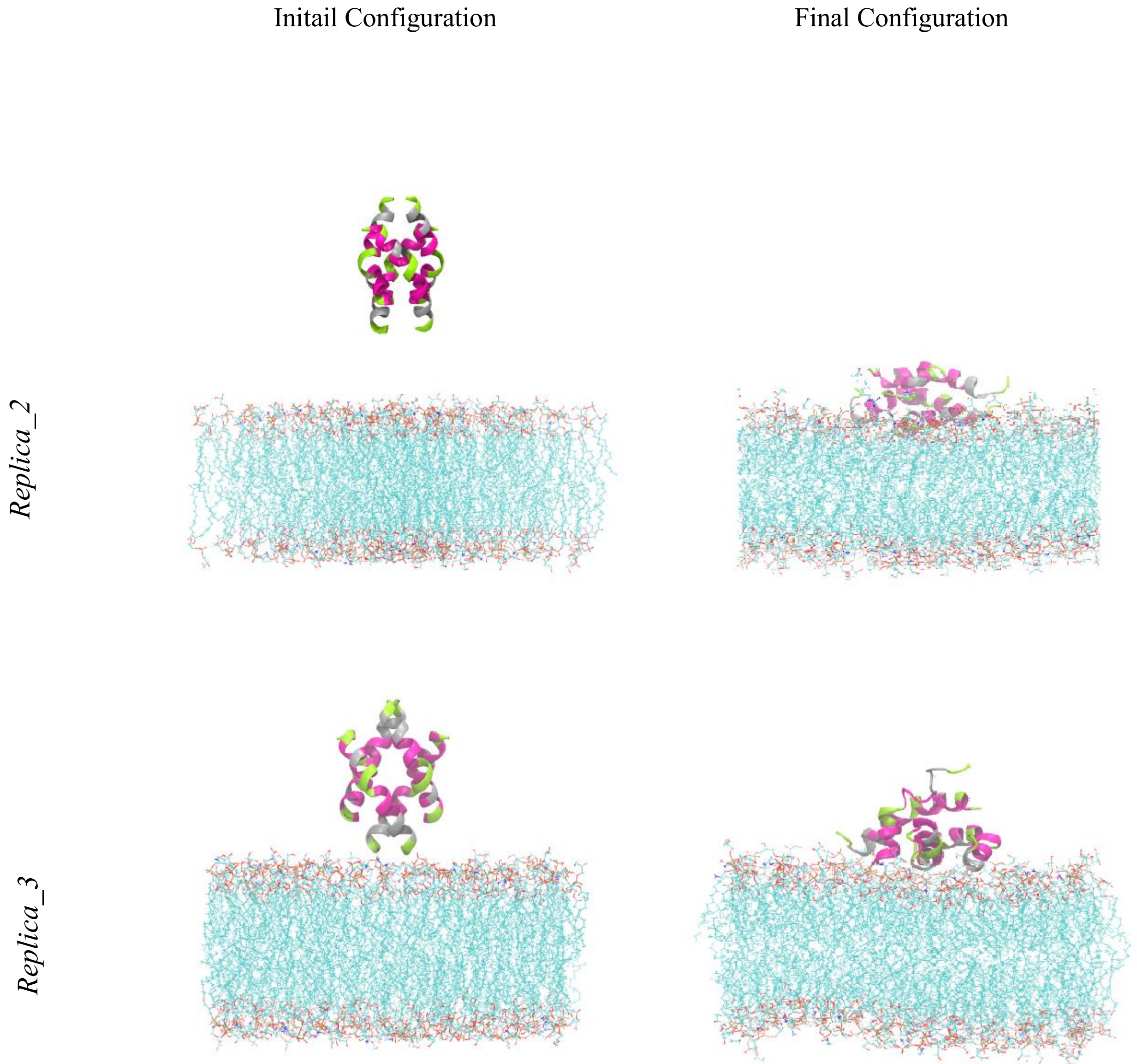

Figure S3: Initial and final configurations for *Replica_2* and *Replica_3* for 4MDP1 systems.

Table S1: The averaged population of the secondary structure (errors) content from DSSP analysis for each peptide in 1MDP1-Water system and 4MDP1-Water system

Simulated Systems	Structures / Number of Peptide	Coil (%)	Bend (%)	Turn (%)	α-Helix (%)	5-Helix (%)	3-Helix (%)
**1 MDP1**	MDP1	28.72 (± 6.97)	5.06 (± 1.48)	9.57 (± 1.53)	55.06 (± 6.95)	0.13 (± 0.04)	1.46 (± 0.37)
**4 MDP1**	MDP1_1	21.39 (±1.23)	4.59 (±0.80)	3.11 (±0.88)	69.89 (±0.61)	0.52 (±0.46)	0.51 (±0.22)
	MDP1_2	14.13 (±1.34)	4 (±0.55)	3.68 (±0.13)	73.71 (±1.86)	0.03 (±0.02)	4.44 (±2.08)
	MDP1_3	10.45 (±0.80)	0.64 (±0.10)	15.8 (±1.83)	70.75 (±1.99)	2.03 (±1.51)	0.33 (±0.10)
	MDP1_4	13.26 (±1.20)	0.67 (±0.21)	4.63 (±0.87)	79.68 (±0.86)	0.17 (±0.15)	1.59 (±0.85)

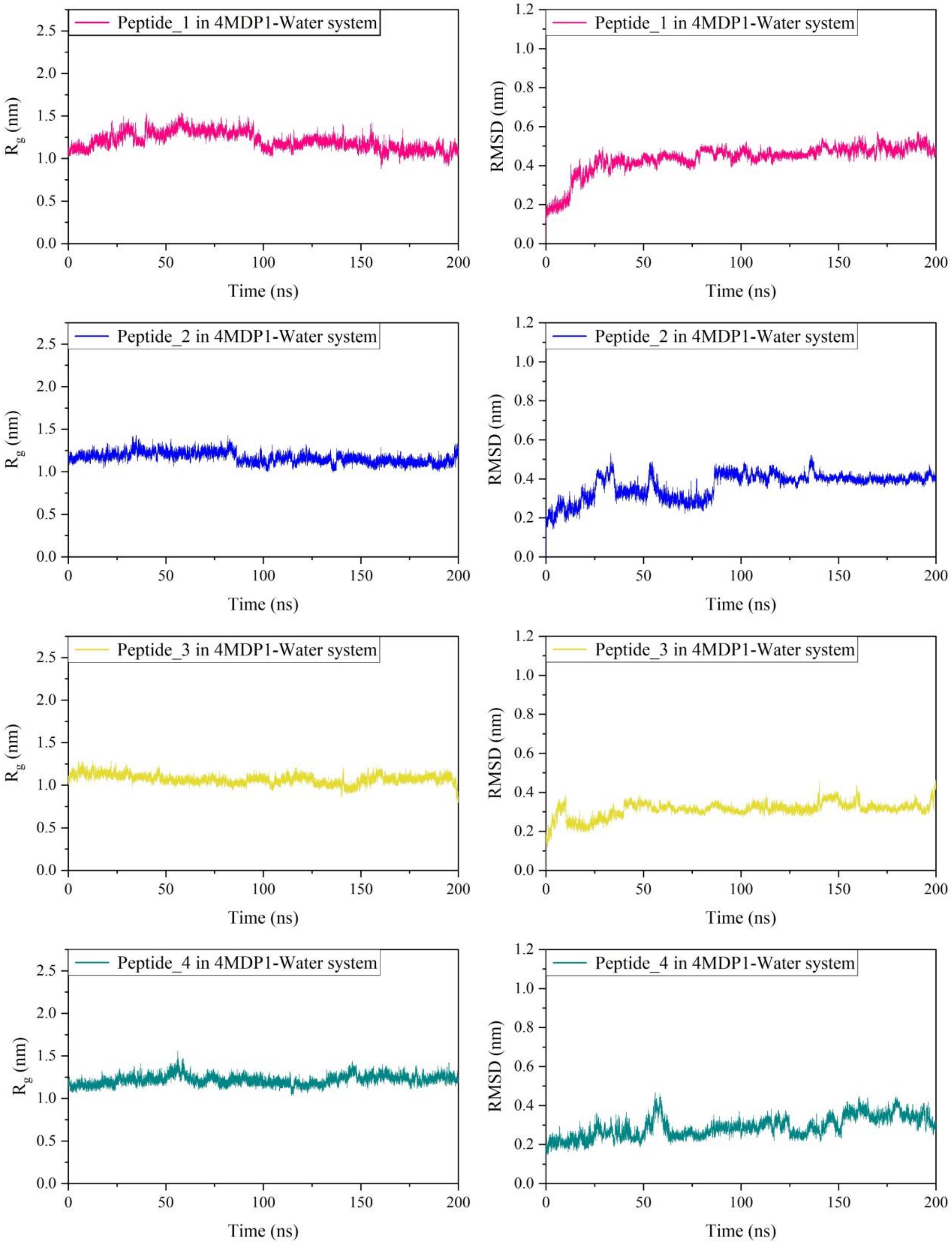

Figure S4: Root mean square deviation (RMSD) and radius of gyration (Rg) of each peptide in 4MDP1-water system as a function of time.

Equilibration Procedures:

NVT-1 (Canonical Ensemble): During this equilibration stage, phosphorus atoms in all phospholipids were restrained with a force constant of 1000 kJ/mol. Additionally, all heavy atoms in peptides were restrained with a force constant of 4000 kJ/mol. Simulations were conducted for 1 ns under these restraints to achieve temperature equilibrium.

NVT-2 (Canonical Ensemble): After achieving temperature equilibrium in NVT-1, once again, phosphorus atoms in all phospholipids were restrained with a force constant of 400 kJ/mol. Additionally, all heavy atoms in peptides were restrained with a force constant of 2000 kJ/mol. Simulations were conducted for 1 ns under these restraints, and temperature equilibrium was reassessed.

NPT-1 (Isothermal–Isobaric Ensemble): During this equilibration stage, phosphorus atoms in all phospholipids were restrained with a force constant of 400 kJ/mol. Additionally, all heavy atoms in peptides were restrained with a force constant of 1000 kJ/mol. Simulations were conducted for 1 ns.

NPT-2 (Isothermal–Isobaric Ensemble): After the first equilibration step in this ensemble, once again, phosphorus atoms in all phospholipids were restrained with a force constant of 200 kJ/mol. Additionally, all heavy atoms in peptides were restrained with a force constant of 500 kJ/mol. Simulations were conducted for 5 ns.

NPT-3 (Isothermal–Isobaric Ensemble): After the second equilibration step in this ensemble, once again, phosphorus atoms in all phospholipids were restrained with a force constant of 40 kJ/mol. Additionally, all heavy atoms in peptides were restrained with a force constant of 200 kJ/mol. Simulations were conducted for 5 ns.

NPT-4 (Isothermal–Isobaric Ensemble): After the third equilibration step in this ensemble, all heavy atoms in peptides were restrained with a force constant of 50 kJ/mol. Simulations were conducted for 5 ns.

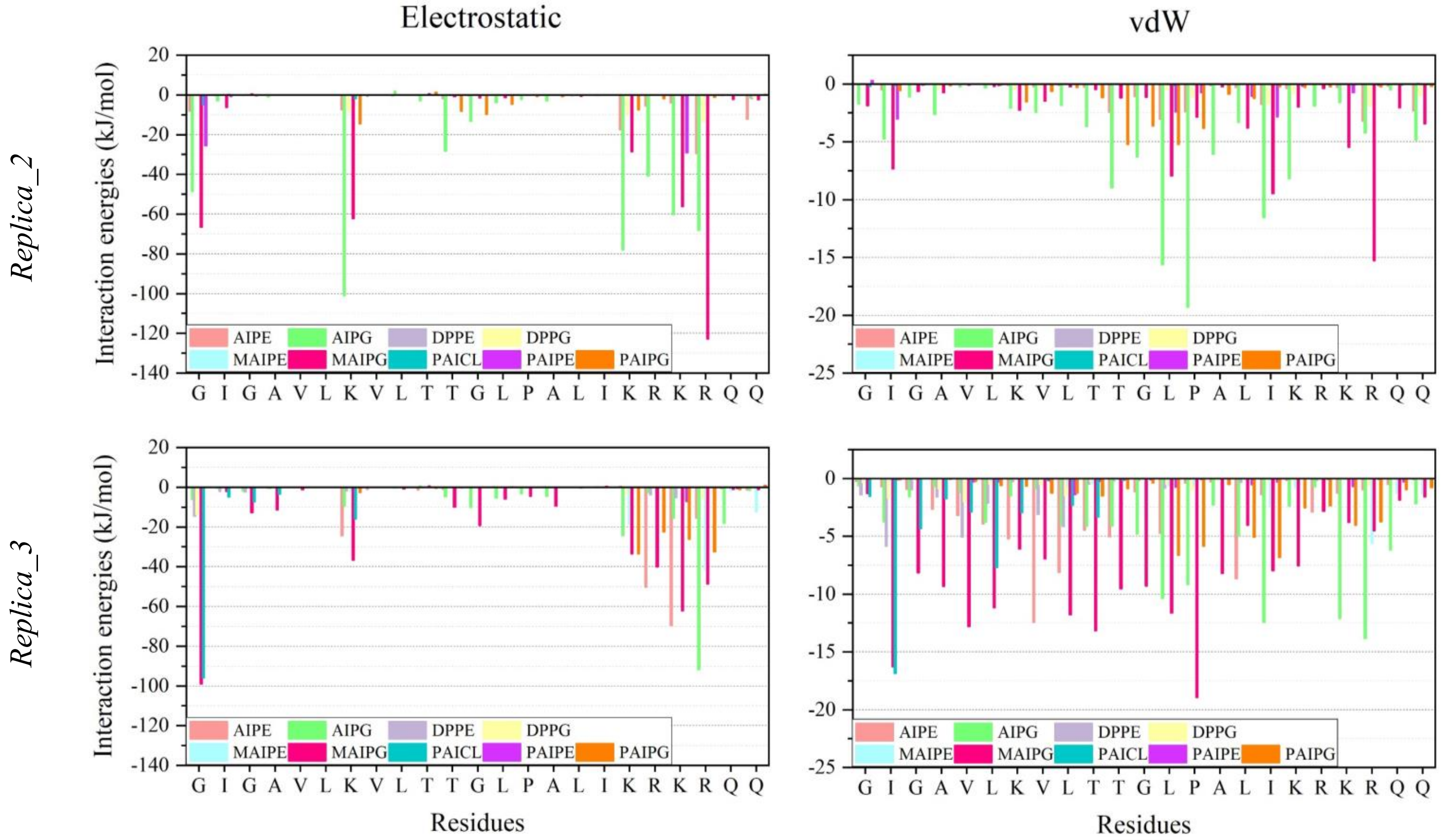

Figure S5: Interaction energies between peptides and different phospholipids for *Replica*_2 and *Replica_3* for 1MDP1 systems.

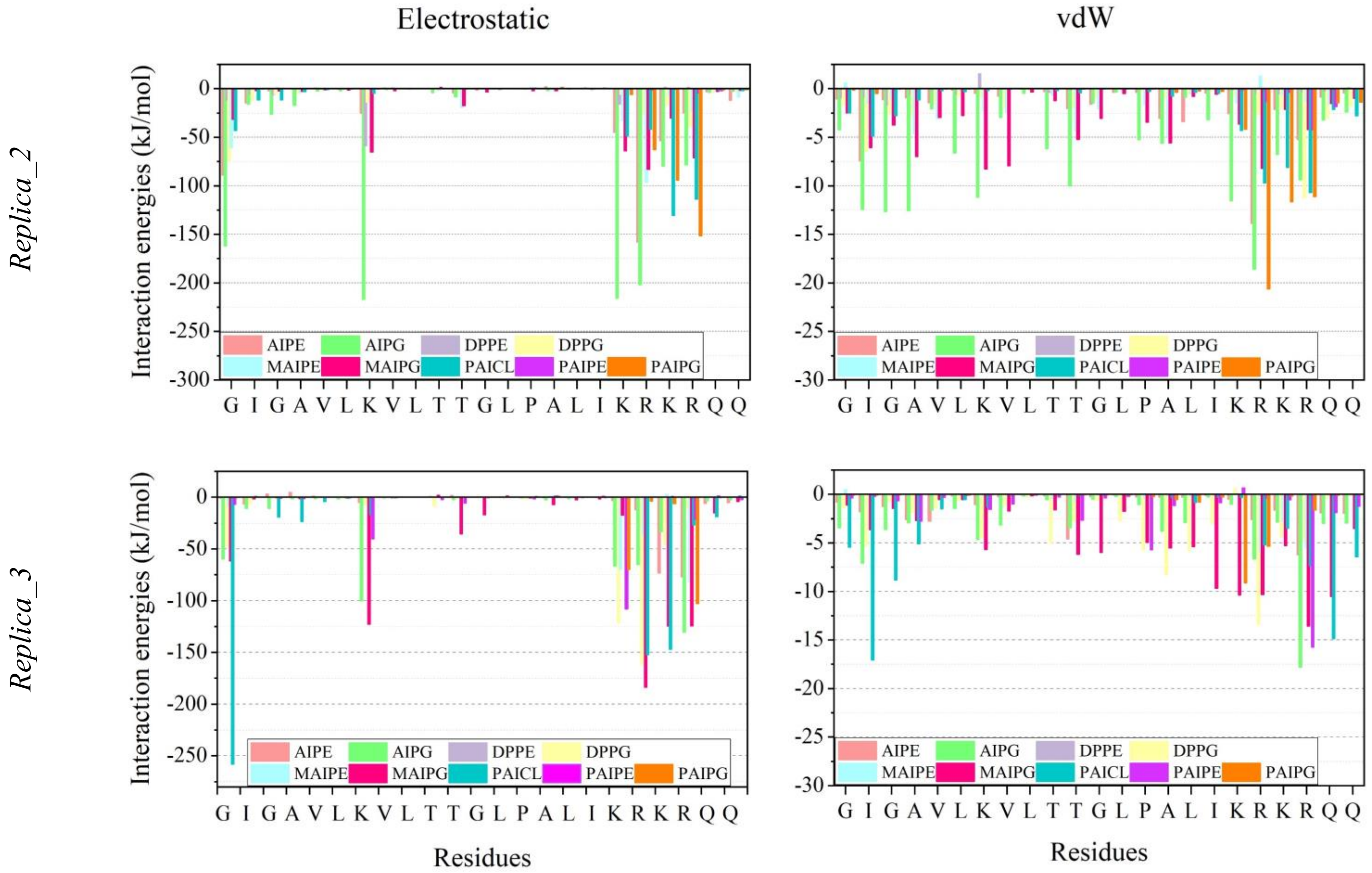

Figure S6: Interaction energies between peptides and different phospholipids for *Replica*_2 and *Replica_3* for 4MDP1 systems.

Table S2: Net contribution (first row) and normalized contribution (second row) of each of the 9 phospholipids to the total electrostatic and vdW interaction energy, expressed as a percentage for the two systems 1MDP1 and 4MDP1.*

Phospholipids / Type of interaction	AIPE	AIPG	DPPE	DPPG	MAIPE	MAIPG	PAICL	PAIPE	PAIPG
1MDP1-Elec	0.37 (±0.54)	15.68 (±5.75)	0.13 (±0.15)	1.51 (±1.11)	4.77 (±2.38)	47.77 (±8.69)	25.13 (±4.74)	0.07 (±0.12)	4.56 (±3.31)
Normalized	0.47 (±0.68)	8.02 (±2.94)	0.59 (±0.71)	2.98 (±2.20)	7.32 (±3.64)	29.98 (±5.46)	43.37 (±8.19)	0.26 (±0.41)	7 (±5.08)
1MDP1-vdW	3.77 (±1.28)	18.20 (±4.64)	0.94 (±0.74)	2.78 (±1.57)	11.08 (±4.19)	44.40 (±5.56)	13.27 (±2.14)	1.08 (±0.39)	4.47 (±2.40)
Normalized	4.64 (±1.57)	9.11 (±2.32)	4.23 (±3.33)	5.37 (±3.03)	16.63 (±6.29)	27.26 (±3.41)	22.41 (±3.61)	3.65 (±1.32)	6.71 (±3.60)
4MDP1-Elec	6.37 (±2.59)	21.78 (±3.70)	0.74 (±0.29)	4.84 (±1.73)	7.00 (±2.45)	33.53 (±4.65)	10.93 (±3.07)	0.26 (±0.22)	14.54 (±3.25)
Normalized	7.55 (±3.06)	10.51 (±1.79)	3.23 (±1.27)	9.01 (±3.23)	10.14 (±3.55)	19.86 (±2.76)	17.8 (±5.01)	0.85 (±0.71)	21.05 (±4.7)
4MDP1-vdW	6.1 (±2.43)	19.40 (±4.49)	0.55 (±0.17)	7.52 (±2.29)	10.99 (±4.34)	32.37 (±5.91)	6.96 (±1.95)	0.27 (±0.18)	15.82 (±3.77)
Normalized	7.02 (±2.79)	9.07 (±2.10)	2.32 (±0.72)	13.56 (±4.14)	15.42 (±6.08)	18.58 (±3.39)	10.99 (±3.08)	0.85 (±0.57)	22.19 (±5.29)

*All results were obtained from the 600-1200ns interval accompanied with errors.

Table S3: Total electrostatic and vdW interaction energy values of peptide residues for the 1MDP1 and 4MDP1 systems.*

Interactions / Residues	**Electrostatic interactions (kJ/mol)**		**vdW interactions (kJ/mol)**	
	**1MDP1**	**4MDP1**	**1MDP1**	**4MDP1**
G_1	-19.34 (±1.60)	-467.41 (±23.23)	-23.7 (±0.62)	-14.28 (±1.87)
I_2	-8.47 (±1.81)	-65.83 (±5.45)	-46.29 (±1.04)	-45.05 (±3.28)
G_3	-23.25 (±2.19)	-63.42 (±7.88)	-16.26 (±0.41)	-27.65 (±2.03)
A_4	-9.2 (±1.30)	-36.43 (±1.55)	-18.52 (±0.75)	-36.16 (±0.34)
V_5	0.6 (±0.10)	-6.4 (±0.51)	-27.98 (±0.22)	-12.56 (±1.50)
L_6	0.37 (±0.16)	-7.04 (±0.73)	-32.27 (±0.64)	-15.29 (±1.54)
K_7	-128.49 (±12.65)	-392.78 (±22.15)	-21.15 (±0.69)	-35.05 (±2.09)
V_8	-0.47 (±0.14)	-1.91 (±0.73)	-26.99 (±1.19)	-24.4 (±3.38)
L_9	0.19 (±0.23)	0.14 (±0.07)	-32.29 (±0.45)	-1.53 (±0.22)
T_10	-4.2 (±0.75)	-19.16 (±6.80)	-26.47 (±0.58)	-11.28 (±1.28)
T_11	-12.45 (±1.64)	-38.32 (±3.76)	-19.07 (±1.11)	-26.28 (±2.50)
G_12	-0.02 (±0.16)	-0.14 (±0.10)	-12.95 (±0.20)	-4.78 (±0.45)
L_13	1.59 (±0.09)	-0.19 (±0.07)	-33.74 (±0.40)	-1.65 (±0.20)
P_14	1.12 (±0.14)	-3.15 (±0.84)	-30.49 (±0.40)	-12.4 (±1.88)
A_15	1.29 (±0.50)	-4.32 (±0.24)	-16.16 (±0.31)	-15.81 (±0.63)
L_16	0.72 (±0.19)	1.25 (±0.16)	-39.03 (±0.40)	-5.12 (±0.50)
I_17	2.88 (±0.27)	-23.27 (±4.36)	-39.88 (±0.18)	-14.63 (±0.73)
K_18	-228.82 (±15.98)	-370.3 (±24.06)	-31.63 (±1.22)	-29.42 (±1.44)
R_19	-189.32 (±6.81)	-746.96 (±37.94)	-47.84 (±0.85)	-77.45 (±2.10)
K_20	-181.25 (±4.98)	-365.34 (±27.05)	-25.34 (±0.77)	-29.13 (±2.58)
R_21	-161.09 (±6.46)	-500.9 (±28.01)	-28.6 (±1.01)	-54.22 (±2.10)
Q_22	-32.15 (±5.72)	-45.58 (±6.25)	-11.48 (±1.50)	-30.86 (±5.42)
Q_23	-13.42 (±2.01)	-26.28 (±12.85)	-8.14 (±1.05)	-10.74 (±1.92)

*All results were obtained from the 600-1200ns interval accompanied with errors.

Table S4: The absolute changes of CH order parameter for the carbon atoms of the SN1 and SN2 chains of each phospholipid in the 1MDP1 and 4MDP1 systems, relative to the pure system.

Phospholipids / Systems	AIPE	AIPG	DPPE	DPPG	MAIPE	MAIPG	PAIPE	PAIPG	PAICL_1	PAICL_2	Sum
**1MDP1 (SN1)**	0.19	0.12	0.22	0.07	0.05	0.13	0.19	0.11	0.19	0.21	1.48
**1MDP1 (SN2)**	0.21	0.09	0.18	0.10	0.12	0.14	0.10	0.07	0.04	0.07	1.12
**1MDP1 (SN1+SN2)**	0.40	0.22	0.41	0.17	0.17	0.27	0.29	0.18	0.23	0.28	2.62
**4MDP1 (SN1)**	0.03	0.02	0.10	0.11	0.08	0.11	0.90	0.04	0.07	0.18	1.64
**4MDP1 (SN2)**	0.07	0.07	0.17	0.08	0.11	0.03	0.57	0.07	0.21	0.05	1.43
**4MDP1 (SN1+SN2)**	0.10	0.09	0.27	0.18	0.19	0.14	1.47	0.11	0.28	0.23	3.06

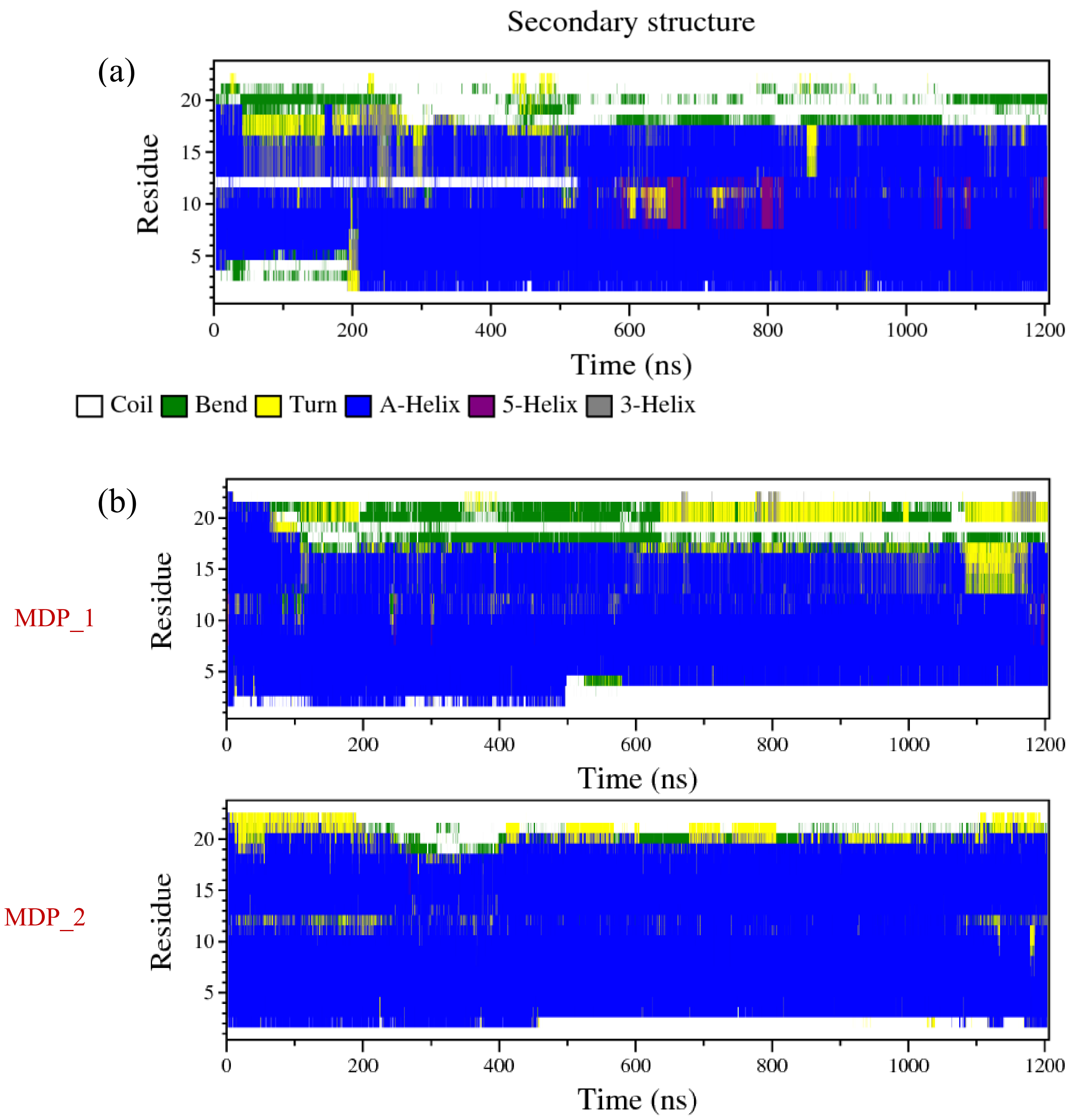

Secondary structure
(a)
Residue
Time (ns)
Coil
Bend
Turn
A-Helix
5-Helix
3-Helix
(b)
MDP_1
MDP_2

MDP_3

MDP_4

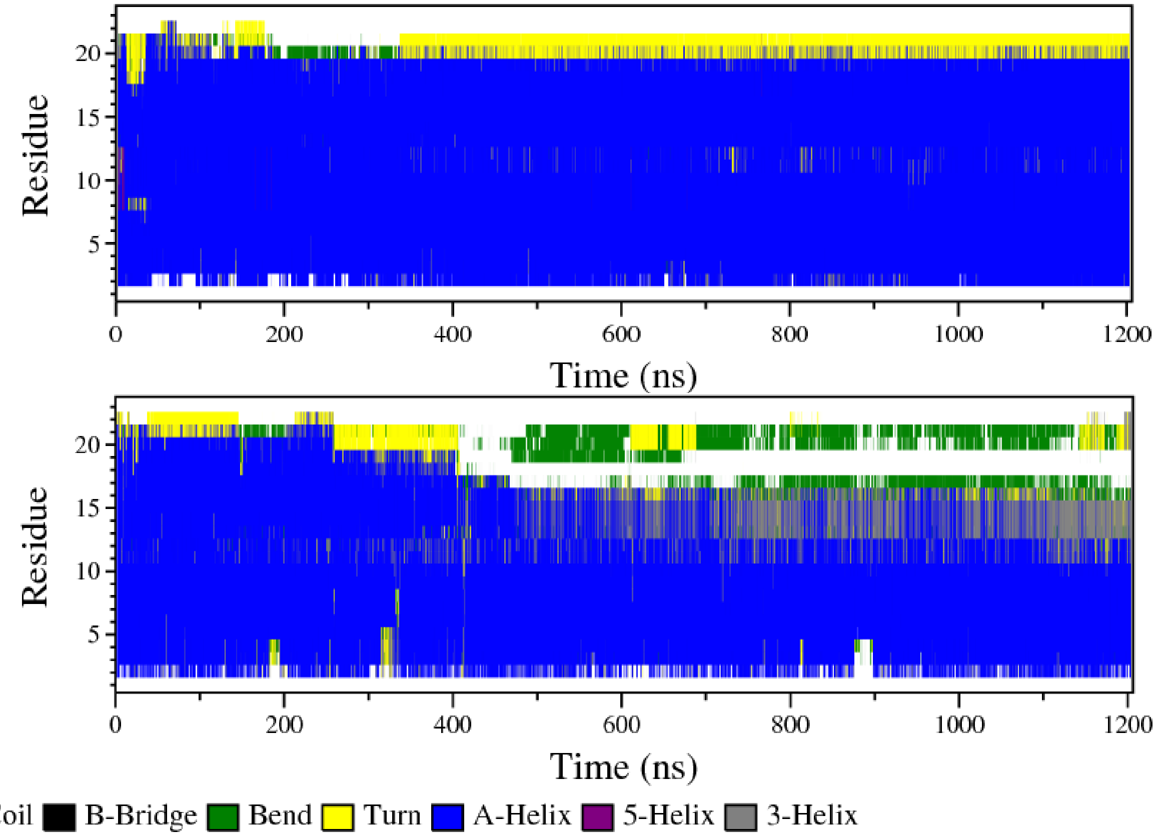

Figure S7: Secondary structures of MDP1 molecules (a) in 1 MDP1, (b) in 4 MDP1systems

Replica_2 in 1MDP1 system:

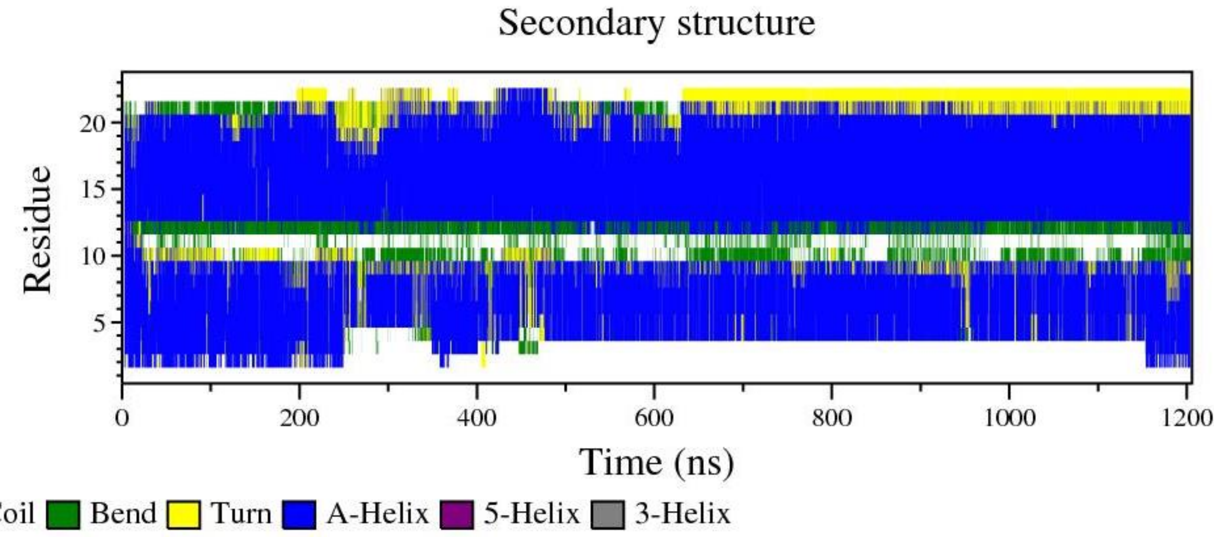

Repelica_3 in 1MDP1 system:

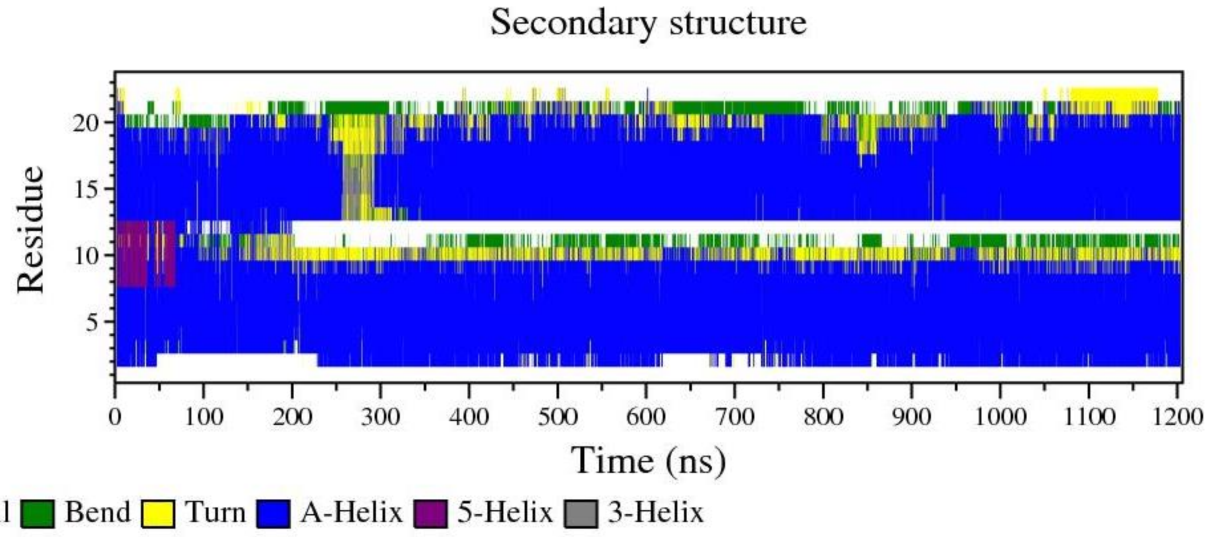

Figure S8: Secondary structures of MDP1 molecules for 1MDP1 systems, *Replica_2* and *Replica_3*.

Replica_2 in 4MDP1 system:

MDP_1

MDP_2

MDP_3

MDP_4

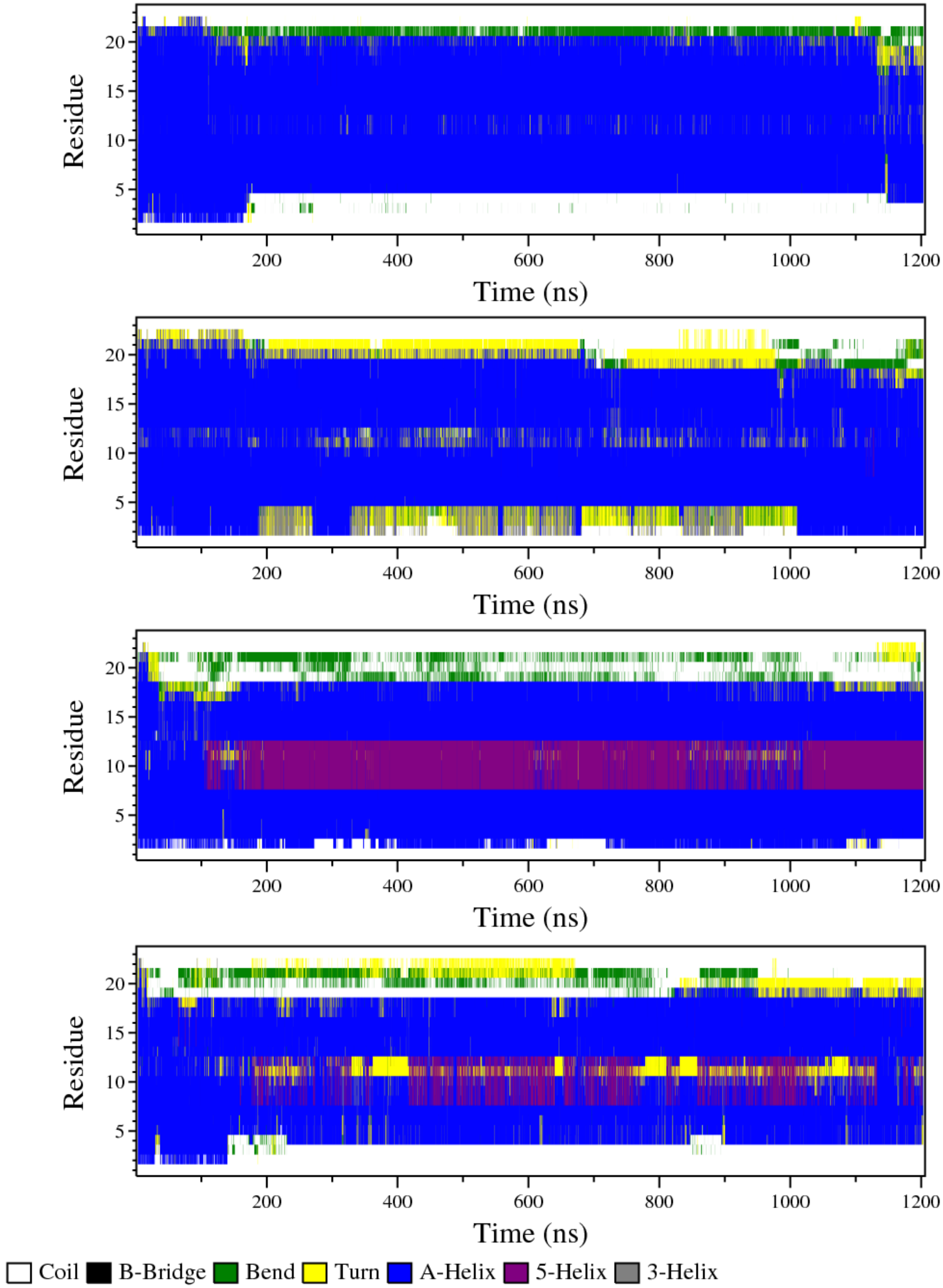

Repelica_3 in 4MDP1 system:

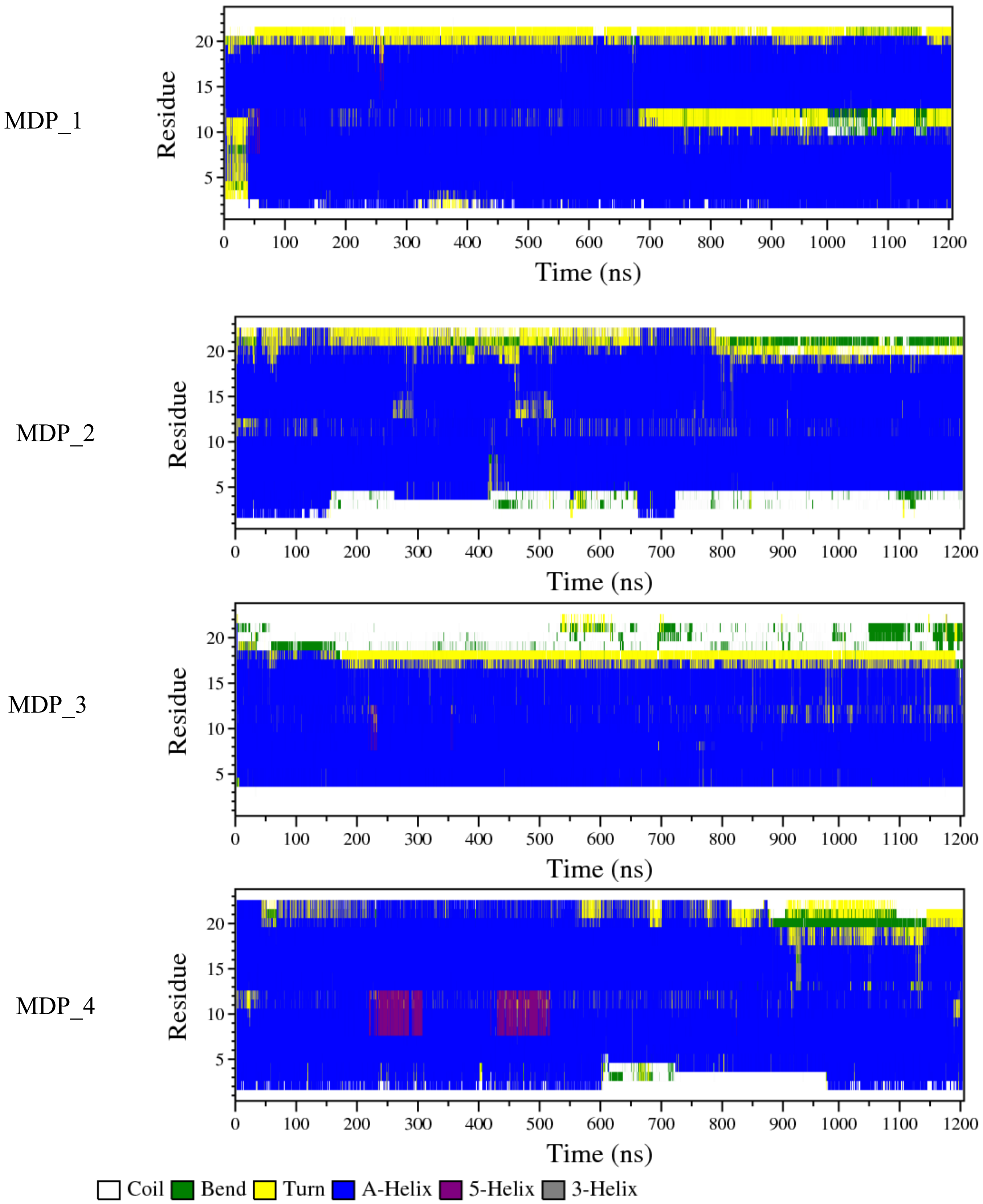

Figure S9: Secondary structures of MDP1 molecules for 4MDP1 systems, *Replica_2* and *Replica_3*.

Table S5: Contribution of each amino acid to electrostatic and van der Waals interactions with 1MDP1 and 4MDP1 systems accompanied with errors.

Interaction energies / Components	Coul-1MDP1	Interaction energies / Components	vdW-1MDP1	Interaction energies / Components	Coul-4MDP1	Interaction energies / Components	vdW-4MDP1
**R-MAIPG**	-251.52 (±26.11)	**L-MAIPG**	-71.24 (±5.62)	**R-MAIPG**	-386.73 (±44.42)	**R-MAIPG**	-47 (±8.37)
**K-PAICL**	-198.32 (±22.02)	**R-MAIPG**	-37.44 (±2.54)	**K-MAIPG**	-322.63 (±47.60)	**K-MAIPG**	-27.21 (±5.86)
**K-MAIPG**	-187.2 (±48.66)	**K-MAIPG**	-29.91 (±4.85)	**R-AIPG**	-296.45 (±34.84)	**R-AIPG**	-26.65 (±5.85)
**K-AIPG**	-87.42 (±23.73)	**K-PAICL**	-28.01 (±3.66)	**K-AIPG**	-276.03 (±37.76)	**V-PAIPE**	-22.09 (±3.55)
**R-PAICL**	-42.8 (±20.37)	**V-MAIPG**	-27.29 (±6.71)	**G-MAIPG**	-250.13 (±49.27)	**K-AIPG**	-21.98 (±2.99)
**K-MAIPE**	-30.48 (±10.90)	**G-MAIPG**	-24.37 (±4.02)	**K-PAIPG**	-215.28 (±24.88)	**A-AIPG**	-19.99 (±3.02)
**R-AIPG**	-26.13 (±16.39)	**I-MAIPG**	-21.52 (±2.23)	**R-PAIPE**	-207.22 (±39.72)	**K-PAIPG**	-19.47 (±3.84)
**Q-MAIPG**	-24.66 (±5.82)	**A-MAIPG**	-21.45 (±3.01)	**R-PAICL**	-153.31 (±36.80)	**K-DPPG**	-17.89 (±3.90)
**K-PAIPG**	-24.63 (±15.13)	**L-AIPG**	-20.99 (±4.09)	**K-DPPG**	-124.39 (±43.36)	**I-MAIPG**	-16.81 (±3.24)
**G-AIPG**	-22.57 (±8.02)	**I-AIPG**	-20.71 (±4.91)	**G-PAICL**	-105.81 (±28.76)	**T-PAIPG**	-16.48 (±3.13)
**K-DPPG**	-12.73 (±8.56)	**R-PAICL**	-18.94 (±2.50)	**G-PAIPE**	-99.03 (±15.18)	**T-AIPG**	-15.22 (±3.98)
**Q-PAICL**	-11.07 (±3.91)	**I-PAICL**	-17.23 (±1.35)	**K-PAICL**	-84.75 (±22.64)	**V-AIPG**	-14.53 (±3.38)
**R-PAIPG**	-10.71 (±10.53)	**T-AIPG**	-15.32 (±2.74)	**R-DPPG**	-76.22 (±22.76)	**G-MAIPG**	-12.15 (±4.99)
**R-MAIPE**	-8.27 (±6.04)	**T-MAIPG**	-13.59 (±1.64)	**G-AIPG**	-59.77 (±22.05)	**I-PAIPE**	-11.97 (±2.79)
**T-AIPG**	-7.79 (±2.91)	**P-MAIPG**	-12.43 (±1.46)	**R-DPPE**	-56.03 (±26.54)	**R-DPPG**	-11.14 (±3.94)
**G-MAIPG**	-7.71 (±2.53)	**G-AIPG**	-12.11 (±2.95)	**K-MAIPE**	-45.29 (±17.94)	**G-AIPG**	-10.86 (±2.73)
**G-PAIPG**	-6.65 (±4.49)	**L-MAIPE**	-12.1 (±2.84)	**K-PAIPE**	-33.66 (±12.40)	**A-PAICL**	-10.3 (±1.58)
**I-AIPG**	-6.33 (±2.67)	**V-AIPG**	-10.2 (±3.99)	**I-MAIPG**	-29.14 (±7.59)	**I-PAICL**	-10.24 (±3.91)
**T-MAIPG**	-4.87 (±1.17)	**T-MAIPE**	-9.96 (±3.39)	**R-PAIPG**	-28.68 (±6.89)	**A-PAIPE**	-8.7 (±1.76)
**A-AIPG**	-4.81 (±1.83)	**I-MAIPE**	-9.61 (±2.72)	**I-PAIPE**	-25.9 (±22.13)	**V-PAICL**	-8.53 (±1.59)
**Q-MAIPE**	-3.64 (±2.42)	**R-AIPG**	-9.29 (±3.08)	**R-MAIPE**	-25.42 (±7.82)	**R-PAIPE**	-8.4 (±2.31)
**K-AIPE**	-3.52 (±3.67)	**Q-MAIPG**	-9.27 (±1.54)	**R-AIPE**	-24.74 (±4.06)	**I-AIPG**	-8.4 (±2.39)
**G-MAIPE**	-2.84 (±1.78)	**K-AIPG**	-7.79 (±2.10)	**A-PAICL**	-23.99 (±5.47)	**P-AIPG**	-7.99 (±2.15)
**T-MAIPE**	-2.51 (±1.05)	**V-MAIPE**	-7.53 (±2.63)	**Q-DPPE**	-22.99 (±15.34)	**A-MAIPG**	-7.58 (±3.85)
**Q-AIPG**	-1.92 (±0.97)	**L-AIPE**	-7.23 (±1.67)	**K-AIPE**	-21.32 (±14.24)	**G-PAIPE**	-7.53 (±3.20)
**G-DPPG**	-1.82 (±1.63)	**V-AIPE**	-7.09 (±3.17)	**T-AIPG**	-20.27 (±7.58)	**R-DPPE**	-7.51 (±0.88)
**A-PAIPG**	-1.77 (±1.19)	**L-PAIPG**	-6.9 (±3.31)	**A-AIPG**	-17.32 (±4.19)	**R-PAICL**	-7.36 (±2.62)
**I-PAIPG**	-1.16 (±0.89)	**P-AIPG**	-6.8 (±1.74)	**T-PAIPG**	-14.04 (±5.70)	**G-PAICL**	-7.16 (±1.60)
**K-PAIPE**	-1 (±0.96)	**R-MAIPE**	-6.39 (±3.54)	**Q-MAIPE**	-12.57 (±6.17)	**L-MAIPG**	-6.92 (±1.47)
**R-DPPE**	-0.96 (±0.93)	**Q-PAICL**	-6.1 (±2.00)	**T-DPPG**	-11.66 (±2.94)	**A-PAIPG**	-6.69 (±1.66)
**A-MAIPG**	-0.79 (±1.02)	**K-MAIPE**	-5.95 (±2.95)	**I-PAICL**	-10.97 (±5.71)	**T-DPPG**	-6.29 (±1.63)
**T-PAIPG**	-0.68 (±0.37)	**L-DPPG**	-5.75 (±2.74)	**Q-AIPG**	-8.8 (±6.03)	**T-PAIPE**	-5.9 (±1.60)
**V-MAIPG**	-0.65 (±0.22)	**P-MAIPE**	-5.55 (±3.09)	**Q-MAIPG**	-7.27 (±3.77)	**R-PAIPG**	-5.79 (±3.67)
**I-MAIPE**	-0.52 (±0.68)	**G-MAIPE**	-5.09 (±1.78)	**Q-PAIPE**	-7.25 (±4.16)	**Q-AIPG**	-5.62 (±1.69)
**A-DPPG**	-0.48 (±0.44)	**L-PAICL**	-5.05 (±1.83)	**V-PAICL**	-6.87 (±4.53)	**Q-MAIPG**	-5.45 (±2.00)
**T-AIPE**	-0.47 (±0.28)	**A-AIPG**	-4.82 (±1.75)	**L-MAIPG**	-6.2 (±2.46)	**I-DPPG**	-5.26 (±0.79)
**G-DPPE**	-0.46 (±0.44)	**I-PAIPG**	-4.62 (±2.56)	**K-DPPE**	-6.19 (±2.79)	**K-PAICL**	-5.09 (±2.41)

**G-AIPE**	-0.43 (±0.33)	**G-PAIPG**	-4.3 (±2.58)	**T-MAIPE**	-6.13 (±1.74)	**V-PAIPG**	-5.06 (±1.86)
**T-PAICL**	-0.28 (±0.23)	**L-PAIPE**	-3.78 (±1.43)	**Q-DPPG**	-6.09 (±2.76)	**P-PAIPG**	-4.99 (±1.29)
**A-MAIPE**	-0.22 (±0.19)	**A-MAIPE**	-3.69 (±1.66)	**A-MAIPG**	-4.53 (±2.58)	**P-MAIPG**	-4.34 (±1.05)
**T-DPPG**	-0.17 (±0.11)	**P-PAICL**	-3.65 (±0.79)	**P-AIPG**	-4.47 (±2.17)	**Q-DPPE**	-4.31 (±0.70)
**R-AIPE**	-0.15 (±0.72)	**K-PAIPG**	-3.54 (±2.33)	**I-AIPG**	-4.43 (±3.74)	**V-DPPG**	-4.13 (±0.75)
**Q-PAIPG**	-0.12 (±0.07)	**T-PAIPG**	-3.42 (±1.03)	**T-MAIPG**	-3.94 (±1.89)	**T-MAIPG**	-3.86 (±1.45)
**V-PAIPG**	-0.06 (±0.09)	**I-AIPE**	-3.24 (±0.80)	**P-PAIPG**	-3.09 (±0.57)	**G-PAIPG**	-3.7 (±0.74)
**R-DPPG**	-0.06 (±0.11)	**I-DPPG**	-3.23 (±1.92)	**I-PAIPG**	-2.98 (±1.24)	**I-PAIPG**	-3.31 (±1.90)
**Q-DPPG**	-0.04 (±0.02)	**G-DPPG**	-2.62 (±1.87)	**L-DPPG**	-2.98 (±1.42)	**L-AIPG**	-3.16 (±1.69)
**G-PAIPE**	-0.03 (±0.05)	**Q-AIPG**	-2.06 (±0.73)	**Q-PAIPG**	-2.69 (±1.00)	**Q-DPPG**	-3.12 (±1.35)
**Q-AIPE**	-0.01 (±0.02)	**G-AIPE**	-2.03 (±0.61)	**V-AIPG**	-2.58 (±1.07)	**R-AIPE**	-2.8 (±0.99)
**G-PAICL**	-0.01 (±0.00)	**K-DPPG**	-1.71 (±1.04)	**V-MAIPG**	-2.37 (±0.79)	**L-DPPG**	-2.58 (±1.32)
**V-PAICL**	-0.01 (±0.00)	**T-DPPE**	-1.54 (±1.45)	**G-PAIPG**	-2.08 (±0.94)	**K-PAIPE**	-2.2 (±0.73)
**T-PAIPE**	-0.01 (±0.01)	**T-DPPG**	-1.49 (±0.70)	**T-AIPE**	-1.77 (±1.94)	**V-MAIPG**	-2.14 (±0.79)
**K-DPPE**	0 (±0.02)	**I-PAIPE**	-1.49 (±0.38)	**T-DPPE**	-1.61 (±0.64)	**L-PAIPG**	-1.9 (±0.57)
**P-DPPE**	0 (±0.00)	**A-PAIPG**	-1.46 (±0.94)	**L-PAICL**	-1.55 (±1.16)	**T-MAIPE**	-1.89 (±0.99)
**Q-DPPE**	0 (±0.00)	**A-AIPE**	-1.43 (±0.82)	**A-PAIPG**	-1.1 (±0.56)	**L-PAIPE**	-1.89 (±0.79)
**T-DPPE**	0 (±0.00)	**R-PAIPG**	-1.4 (±1.34)	**L-PAIPG**	-1.1 (±0.15)	**A-DPPG**	-1.79 (±0.56)
**A-PAIPE**	0 (±0.00)	**Q-MAIPE**	-1.13 (±0.73)	**V-PAIPE**	-1.01 (±0.20)	**R-MAIPE**	-1.7 (±0.91)
**Q-PAIPE**	0 (±0.00)	**K-DPPE**	-1.1 (±0.82)	**P-MAIPG**	-1 (±0.67)	**T-PAICL**	-1.6 (±0.96)
**A-DPPE**	0.01 (±0.00)	**L-DPPE**	-1.06 (±0.99)	**L-PAIPE**	-0.87 (±0.30)	**K-DPPE**	-1.47 (±0.75)
**V-DPPG**	0.01 (±0.01)	**T-PAICL**	-0.89 (±0.72)	**Q-AIPE**	-0.61 (±0.26)	**Q-PAIPE**	-1.23 (±0.09)
**R-PAIPE**	0.01 (±0.01)	**T-AIPE**	-0.84 (±0.25)	**Q-PAICL**	-0.29 (±0.23)	**K-MAIPE**	-1.17 (±0.69)
**I-DPPE**	0.02 (±0.01)	**A-DPPG**	-0.8 (±0.55)	**A-PAIPE**	-0.29 (±0.16)	**G-DPPG**	-1.09 (±0.14)
**P-PAIPE**	0.02 (±0.01)	**R-DPPE**	-0.76 (±0.72)	**G-DPPG**	-0.19 (±0.43)	**K-AIPE**	-0.9 (±0.71)
**V-PAIPE**	0.02 (±0.01)	**V-PAIPG**	-0.65 (±0.31)	**T-PAIPE**	-0.17 (±1.40)	**Q-MAIPE**	-0.75 (±0.69)
**P-AIPE**	0.03 (±0.01)	**P-PAIPG**	-0.59 (±0.04)	**P-PAICL**	-0.15 (±0.14)	**T-DPPE**	-0.59 (±0.20)
**L-DPPE**	0.03 (±0.04)	**V-DPPE**	-0.54 (±0.19)	**P-DPPE**	-0.12 (±0.07)	**Q-PAIPG**	-0.54 (±0.16)
**I-DPPG**	0.03 (±0.08)	**V-DPPG**	-0.53 (±0.34)	**G-MAIPE**	-0.12 (±0.14)	**A-MAIPE**	-0.47 (±0.18)
**P-DPPG**	0.05 (±0.02)	**K-AIPE**	-0.44 (±0.27)	**P-MAIPE**	-0.1 (±0.65)	**L-PAICL**	-0.45 (±0.27)
**P-PAICL**	0.05 (±0.07)	**V-PAIPE**	-0.35 (±0.16)	**P-DPPG**	-0.1 (±0.10)	**P-MAIPE**	-0.44 (±0.30)
**I-PAIPE**	0.05 (±0.04)	**R-AIPE**	-0.34 (±0.09)	**L-AIPG**	-0.08 (±0.01)	**P-DPPG**	-0.35 (±0.13)
**P-PAIPG**	0.05 (±0.01)	**P-DPPG**	-0.34 (±0.15)	**A-AIPE**	-0.07 (±0.18)	**V-MAIPE**	-0.35 (±0.24)
**V-AIPG**	0.06 (±0.26)	**A-DPPE**	-0.32 (±0.11)	**I-MAIPE**	-0.06 (±0.15)	**T-AIPE**	-0.31 (±0.14)
**A-AIPE**	0.07 (±0.10)	**A-PAICL**	-0.32 (±0.05)	**L-DPPE**	-0.02 (±0.04)	**Q-PAICL**	-0.27 (±0.07)
**A-PAICL**	0.07 (±0.03)	**T-PAIPE**	-0.31 (±0.10)	**L-AIPE**	-0.02 (±0.01)	**G-MAIPE**	-0.24 (±0.05)
**L-PAICL**	0.07 (±0.14)	**R-DPPG**	-0.28 (±0.14)	**I-DPPE**	-0.01 (±0.15)	**P-PAIPE**	-0.15 (±0.04)
**V-DPPE**	0.08 (±0.09)	**G-PAIPE**	-0.23 (±0.10)	**G-DPPE**	-0.01 (±0.02)	**V-DPPE**	-0.08 (±0.07)
**I-AIPE**	0.1 (±0.07)	**Q-DPPE**	-0.2 (±0.08)	**A-DPPG**	0 (±0.00)	**A-AIPE**	-0.06 (±0.05)
**L-DPPG**	0.15 (±0.11)	**P-PAIPE**	-0.18 (±0.06)	**A-DPPE**	0 (±0.00)	**Q-AIPE**	-0.06 (±0.04)
**I-MAIPG**	0.17 (±0.48)	**Q-PAIPG**	-0.16 (±0.07)	**V-AIPE**	0 (±0.00)	**P-PAICL**	-0.04 (±0.04)
**L-PAIPE**	0.2 (±0.09)	**K-PAIPE**	-0.14 (±0.12)	**V-MAIPE**	0 (±0.00)	**L-MAIPE**	-0.04 (±0.03)

**V-AIPE**	0.22 (±0.09)	**P-AIPE**	-0.12 (±0.02)	**I-AIPE**	0 (±0.00)	**P-AIPE**	-0.03 (±0.03)
**L-MAIPE**	0.24 (±0.30)	**I-DPPE**	-0.09 (±0.08)	**L-MAIPE**	0 (±0.00)	**P-DPPE**	-0.02 (±0.00)
**P-MAIPG**	0.24 (±0.11)	**Q-DPPG**	-0.09 (±0.06)	**P-AIPE**	0 (±0.00)	**I-MAIPE**	-0.02 (±0.01)
**L-PAIPG**	0.26 (±0.21)	**G-PAICL**	-0.08 (±0.03)	**V-DPPG**	0 (±0.00)	**A-DPPE**	-0.01 (±0.00)
**P-MAIPE**	0.31 (±0.17)	**Q-AIPE**	-0.07 (±0.03)	**V-DPPE**	0.01 (±0.01)	**G-AIPE**	-0.01 (±0.00)
**P-AIPG**	0.37 (±0.16)	**G-DPPE**	-0.07 (±0.03)	**P-PAIPE**	0.01 (±0.00)	**V-AIPE**	0 (±0.00)
**L-AIPG**	0.38 (±0.33)	**A-PAIPE**	-0.03 (±0.01)	**A-MAIPE**	0.04 (±0.03)	**I-AIPE**	0 (±0.00)
**V-MAIPE**	0.4 (±0.13)	**R-PAIPE**	-0.03 (±0.01)	**G-AIPE**	0.05 (±0.08)	**G-DPPE**	0 (±0.00)
**L-AIPE**	0.43 (±0.11)	**V-PAICL**	-0.01 (±0.01)	**I-DPPG**	0.08 (±0.06)	**I-DPPE**	0 (±0.00)
**L-MAIPG**	1.16 (±0.49)	**P-DPPE**	0 (±0.00)	**V-PAIPG**	0.57 (±0.39)	**L-AIPE**	0 (±0.00)
**I-PAICL**	2.01 (±0.49)	**Q-PAIPE**	0 (±0.00)	**T-PAICL**	0.67 (±0.52)	**L-DPPE**	0 (±0.00)
